# Study on the properties of mud films derived from naturally weathered red-bed soil under pressureless conditions

**DOI:** 10.1371/journal.pone.0275867

**Published:** 2022-10-10

**Authors:** Yi Gao, Zhen Liu, Cuiying Zhou

**Affiliations:** School of Civil Engineering, Sun Yat-sen University, Guangzhou, China; Tribhuvan University, NEPAL

## Abstract

Mulching film is an important material in ecological restoration, such as desert control, saline alkali land improvement, and slope greening protection. At present, both solid and liquid mulching films are mainly made of man-made materials, which have a great impact on the ecological environment and are expensive. Naturally weathered red-bed soil mud films are being applied in the construction industry because of their advantages of environmental friendliness and low cost. However, at present, film formation needs to occur under pressure, in complex processes, which results in great application limitations, so it is difficult to popularize and apply these natural mud films to the field of ecological restoration. Therefore, exploring high-quality film formation under pressureless conditions is key for the application of naturally weathered red-bed soil mud films in the field of ecological restoration. To solve this problem, this paper takes naturally weathered red-bed soil, without additives; designs and carries out mud film-forming tests of naturally weathered red-bed soil and a comparison test with bentonite under pressureless conditions; tests the influence of mud particle gradation, viscosity and moisture content on the film-forming effect; optimizes the mud film-forming ratio; and further studies the thickness, water resistance, compression resistance. Acid and alkali resistances are tested and compared with international standards. The results show that the naturally weathered red-bed soil can form a mud film under pressureless conditions. The optimal (high-quality) film corresponds to a mud viscosity of 29.6–52.6 Pa·s, water content of 15–35%, and content of particles with particle sizes smaller than 1 mm of 75%-85%. Compared with international standards and bentonite, the naturally weathered red-bed soil mud film meets the ecological restoration standard, has a better water resistance, compression resistance, and acid and alkali resistances than bentonite mud film, and has the advantages of a wide distribution, low cost, simple sample preparation and wide application range. Therefore, weathered red-bed soil mud film is a film technology with obvious comparative advantages that can be introduced into the field of ecological restoration and has broad prospects.

## Introduction

Plastic film is widely used in ecological restoration, such as desert control, saline alkali land improvement, and slope greening protection. Most mulching films commonly used in production are man-made materials that include many chemicals with high costs and environmental pollution potential [1]. Naturally weathered red-bed soil is a widely available material. It is easy to obtain, is inexpensive and does not pollute the environment. Its mud film is widely used in industrial construction and other fields [2–4]. Suitable naturally weathered red-bed soil mud materials will form mud films after appropriate film-forming processes. At present, the film-forming preparation of naturally weathered red-bed soil mud mainly depends on pressurization conditions, which take a long time to achieve. The film-forming process cannot be carried out in the natural environment and thus cannot be applied to the field of ecological restoration [5, 6]. Therefore, it is necessary to study mud films made with naturally weathered red-bed soil, which can form high-quality films under pressureless conditions and can be applied to the field of ecological restoration.

Research on mud film formation has mainly focused on artificial mud film formation and natural mud film formation. In terms of man-made mud film formation, Fritz [7] added polymer additives to bentonite suspensions through laboratory tests so that the mud could be made into 50 mm mud films in high-permeability formations. Luheng [8] found that a polymer drilling mud system has a good rheology and flocculation ability, and a 45 mm mud film was made by this method. Sodium carboxymethyl cellulose (CMC) and polyaluminium chloride are added to a salt water slurry to maintain the slurry viscosity. Compared with bentonite polymers, such slurries can be thinner and better in quality. Menezes et al. [9] studied the effect of CMC on the apparent and plastic viscosity of bentonite dispersion of water-based drilling fluid and made a 40 mm mud film with CMC. Min et al. [10] added sodium CMC to bentonite slurry to adjust the rheological properties such as slurry viscosity so that the slurry can form a higher-quality mud film, but this can only improve the film quality, and the thickness cannot be reduced. Compared with the natural materials of weathered red-bed soil, the raw materials of artificial mud are relatively high in cost. The extensive use of chemical additives in artificial mud will have an impact on the environment. Moreover, natural materials have good compatibility with the environment. Therefore, the effective use of naturally weathered red-bed soil mud to form films is of great significance and practical value. In terms of natural mud film formation, Min et al. [9] and other researchers have shown that three types of mud films will be formed when different mud penetrates into the formation. On this basis, Tien et al. [11] deduced a mud film filtration formula and solution method considering the dynamic change in the mud film and found that the retention of fine particles significantly reduced the permeability of the mud film, thus changing the filtration characteristics of the mud film. Talmon et al. [12] explored the mechanism of thin mud films formed by natural soil through experiments and prepared 35–40 mm mud films. Zhou et al. [13] made a mud film with a thickness of 30–35 mm using naturally weathered red-bed soil under a pressurized environment. However, the closed pressure condition is not easy to construct, and the mud film prepared by this method has a complex formation process and considerable application limitations, so it cannot be applied to the field of ecological restoration. Therefore, exploring high-quality film formation under pressureless conditions is the key problem for the application of naturally weathered red-bed soil mud films in the field of ecological restoration.

Based on this, we selected a widely distributed naturally weathered red-bed soil, without additives; designed and carried out a mud film-forming test of naturally weathered red-bed soil under pressureless conditions and a comparison test with bentonite; tested the influence of mud particle gradation, viscosity and moisture content on the film-forming effect; optimized the mud film-forming ratio; and further studied the thickness, water resistance, compression resistance, and acid and alkali resistance test results, including a comparison with international standards. We preliminarily verify the feasibility and reliability of the application of weathered red-bed soil mud film-forming under pressureless conditions, improve and develop low-cost green naturally weathered red-bed soil materials and film-forming technology, and provide new support for the introduction of weathered red-bed soil mud film into the field of ecological restoration.

## Research contents and methods

Mulching film is an essential material in ecological restoration, such as desert control, saline alkali land improvement, and slope greening protection. At present, the commonly used mulching films are mainly made of man-made materials, with high chemical composition, serious environmental pollution and high cost. Naturally weathered red-bed soil mud film has good compatibility with the environment and is widely distributed in nature. It is widely used in the construction industry. However, at present, film formation must be formed under pressure: the process is complex, and the application has great limitations, so it is difficult to be directly popularized and applied to the field of ecological restoration. Therefore, exploring high-quality film formation under pressureless conditions is one of the key problems in the application of naturally weathered red-bed soil mud films in the field of ecological restoration. To solve this problem, this paper takes naturally weathered red-bed soil as the study material, without additives; designs and carries out film-forming tests of naturally weathered red-bed soil slurries under pressureless conditions and control tests with bentonite; tests the influences of the particle gradation, viscosity and moisture content of weathered red-bed soil slurries on the film-forming effect; and optimizes the slurry film-forming ratio. We further test the water resistance, compression resistance, and acid and alkali resistances of naturally weathered red-bed soil mud film and bentonite mud film, compare them with international standards to verify the feasibility and reliability of weathered red-bed soil mud film-forming under pressureless conditions, and apply them to the field of ecological restoration.

### Mud material preparation

**Fig 1:**
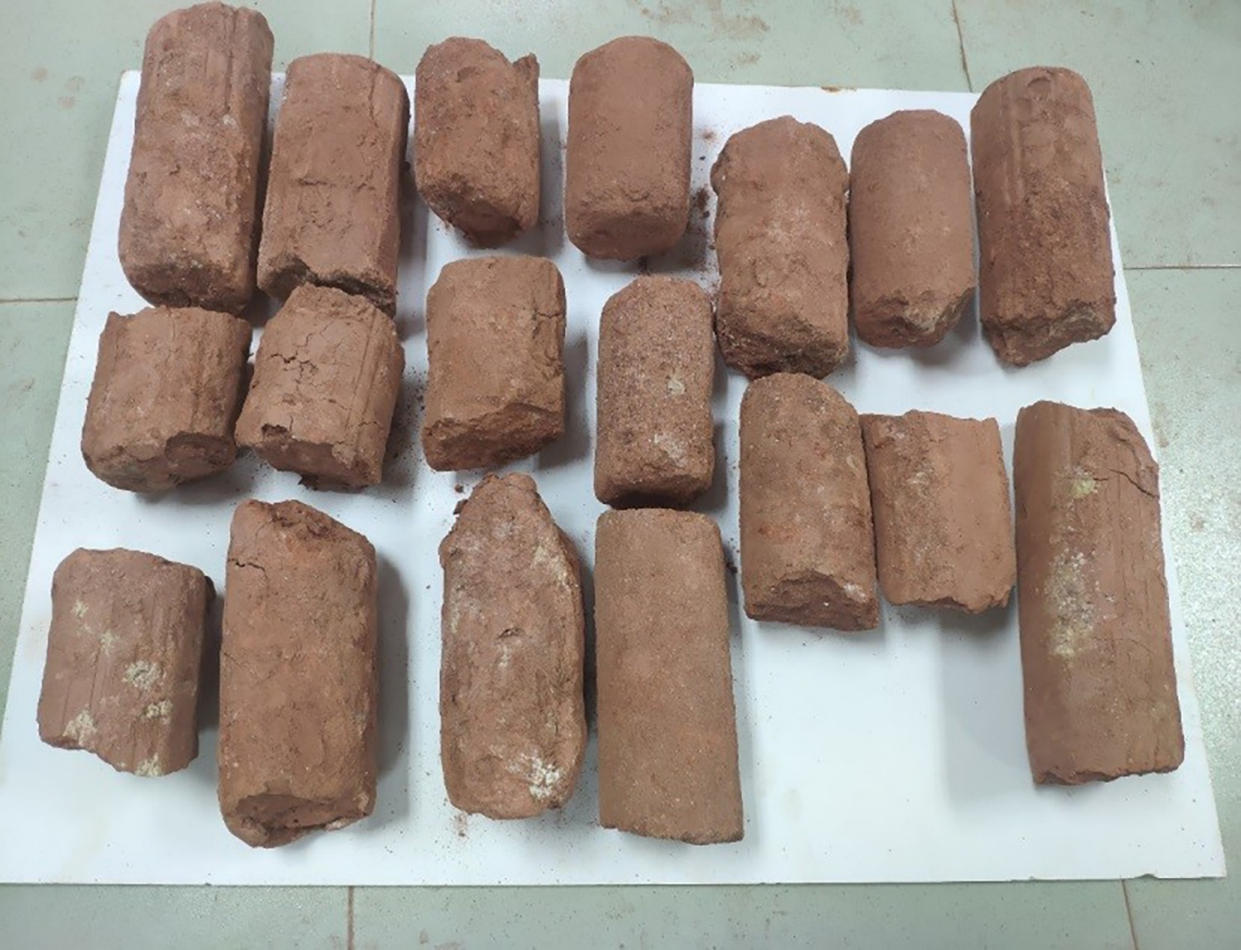
Naturally Weathered Red-Bed Soil Sample (A) No. 1; (B) No. 2; (C) No. 3; (D) No. 4; (E) No. 5; (F) No. 6 (G) No. 7; (H) No. 8; (I) No. 9.

The slurry is made from the weathered red-bed soil common in South China, as shown in Fig 1. The sampling depth is less than 12 m. It is bright red in the natural state and has a high sand content.

The content of weathered red-bed soil with a particle size smaller than 1 mm used in the work is 29%-85%. The nonuniformity coefficient Cu is approximately 6–16, the curvature coefficient is approximately 0.4–1.2, and the soil is well graded. Laser particle size analysis is applied to particles with particle sizes smaller than 0.075 mm: the particle size distribution range is generally 0.416–98.075 μm, and the particle content smaller than 0.416 μm is 0.09%-0.18%. Most particles, accounting for 57%-63%, are distributed in the range of 7–38 μm.

**Fig 2:**
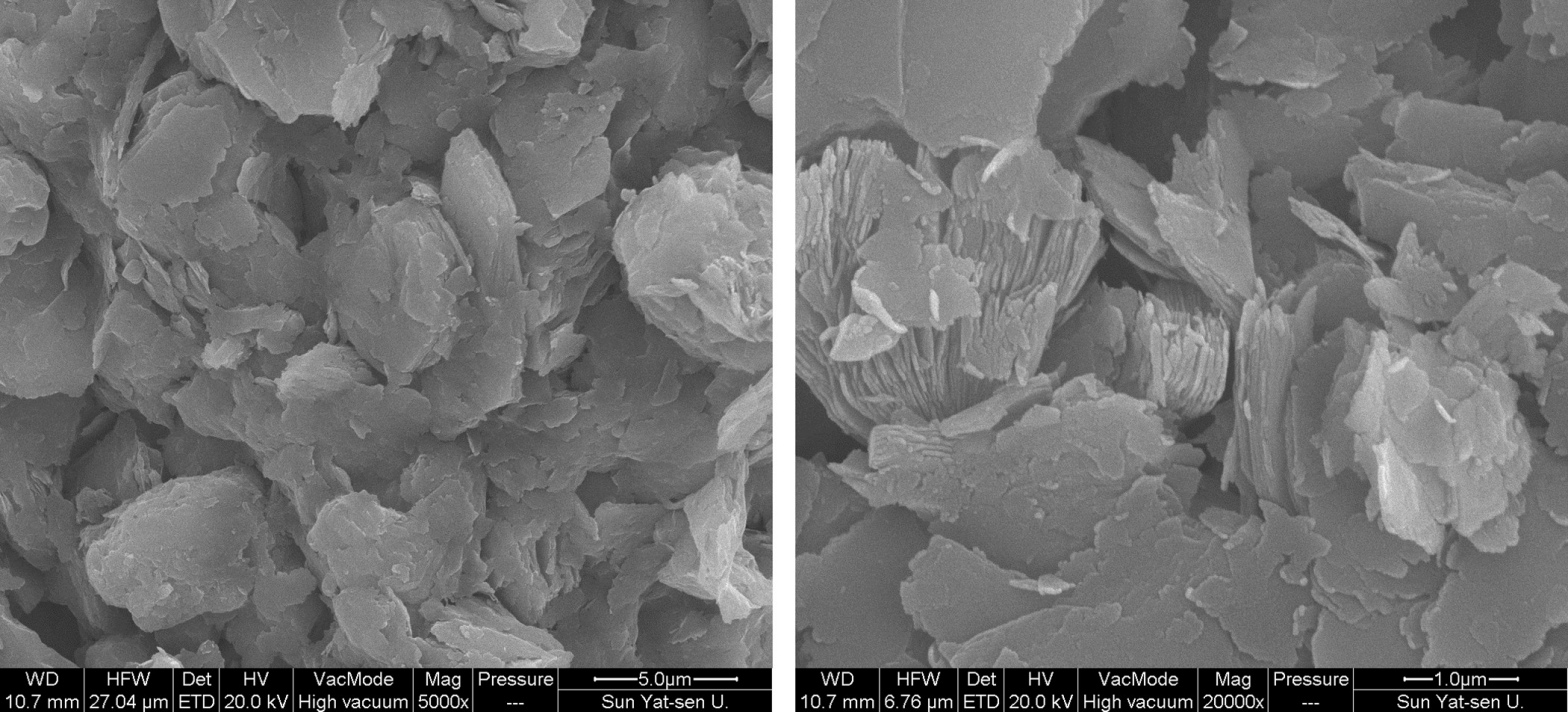
SEM Images of the Weathered Red-Bed Soil (A) Magnified view of 5000 times; (B) Magnified view of 20000 times.

The microstructure scanning electron microscopy (SEM) images of the weathered red-bed soil analysed in this test in the natural state were observed under a field of view enlarged to more than 5000 times. As shown in Fig 2, its microstructure is basically a polygonal sheet structure. The accumulation morphology of the sheet structure is similar to that of a book, arranged closely, mainly in surface-to-surface contact, with pore space between these bodies and other particles, which are evenly distributed. The diameter of the flaky particles and pores is only a few microns. The above characteristics can be summarized as follows: the weathered soil particles in the red layer are accumulated, the thin layers are formed in a continuous and nondirectional arrangement, and the multilayer thin layers are stacked together in parallel to form a thin-layer accumulation structure.

**Fig 3:**
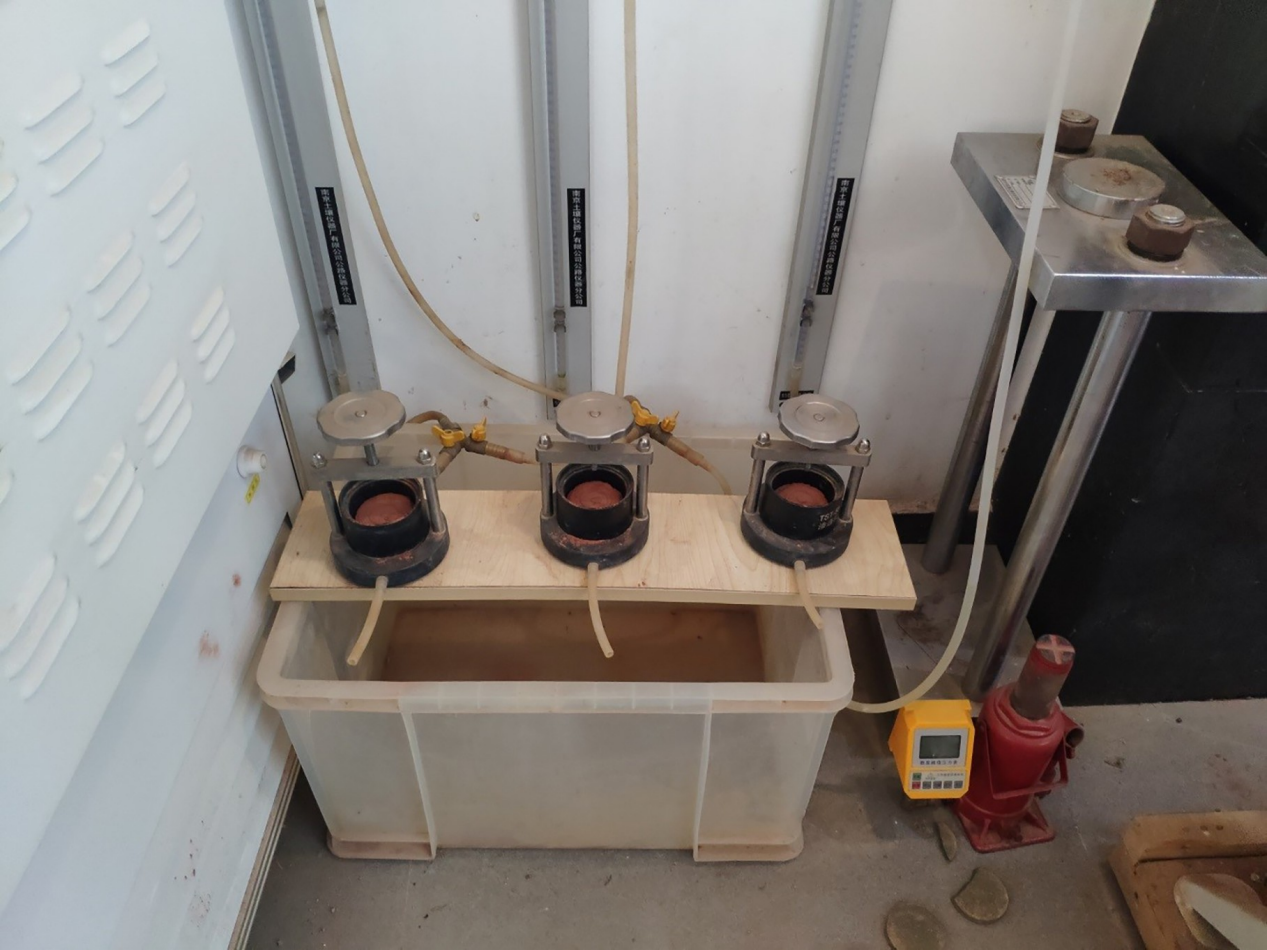
Mud film permeability test.

A TST-55 permeameter is used to carry out the permeability test of the weathered red-bed soil mud film, as shown in Fig 3. A stainless steel ring knife with a diameter of 61.8 mm and a height of 40 mm is used to make the mud film, which is put into the permeameter to carry out the variable head permeability test.

The relative content of each component of the weathered red-bed soil sample is calculated by powder diffraction analysis. The silica content is approximately 66.32%-81.07%. The clay minerals are mainly kaolinite and illite, with a small amount of chlorite and montmorillonite. The kaolinite content is approximately 2.48%-13.36%, illite content is approximately 5.09%-12.11%, chlorite content is approximately 1.32%-4.94%, montmorillonite content is approximately 0.38%-1.83%, anorthite content is approximately 0.5%-7.28%, and haematite content is approximately 1.86%-7.16%.

**Fig 4:**
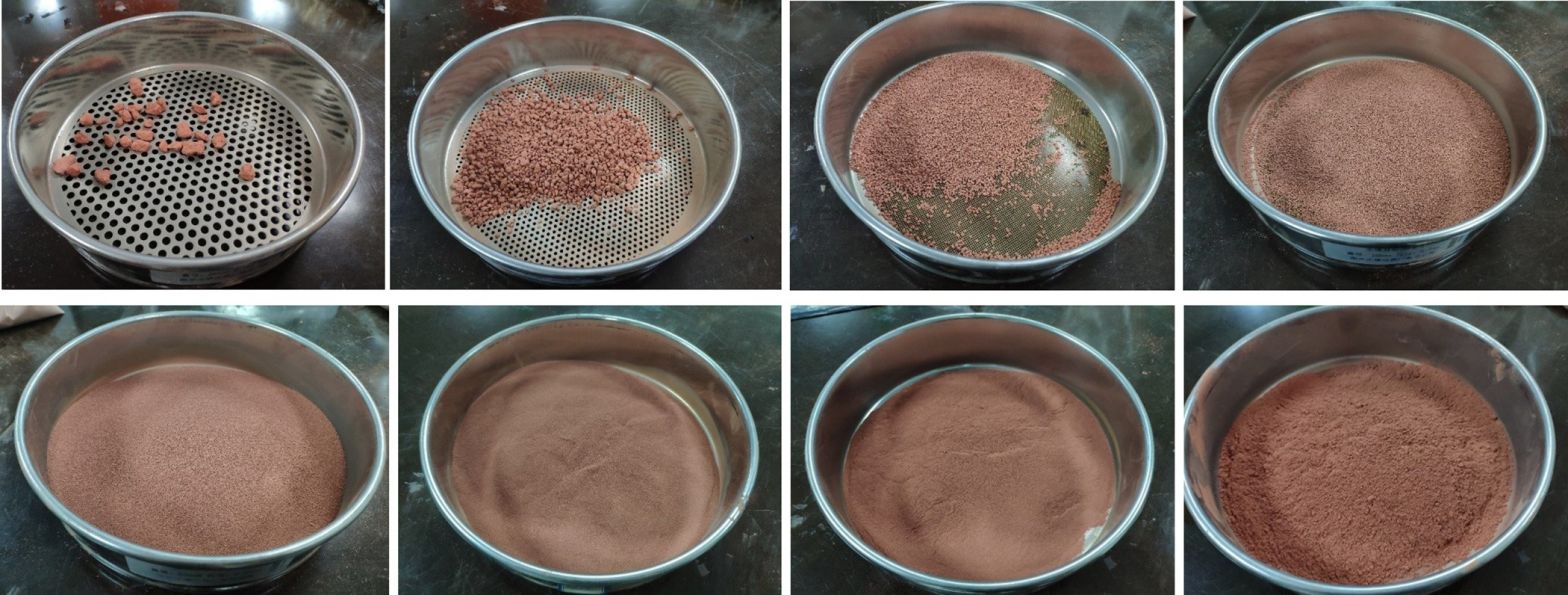
Naturally weathered red-bed soil particles corresponding to each particle size (A) >5 mm; (B) 2–5 mm; (C) 1–2 mm; (D) 0.5–1 mm; (E) 0.25–0.5 mm; (F) 0.1–0.25 mm; (G) 0.075–0.1 mm; (H) <0.075 mm.

The dried weathered red-bed soil particles are poured into a standard sieve and vibrated mechanically for more than 20 min. After the quality of weathered red-bed soil particles of each particle size becomes stable, they are weighed to obtain the particle gradation of the sample, as shown in Fig 4.

### Mud film formation

**Fig 5:**
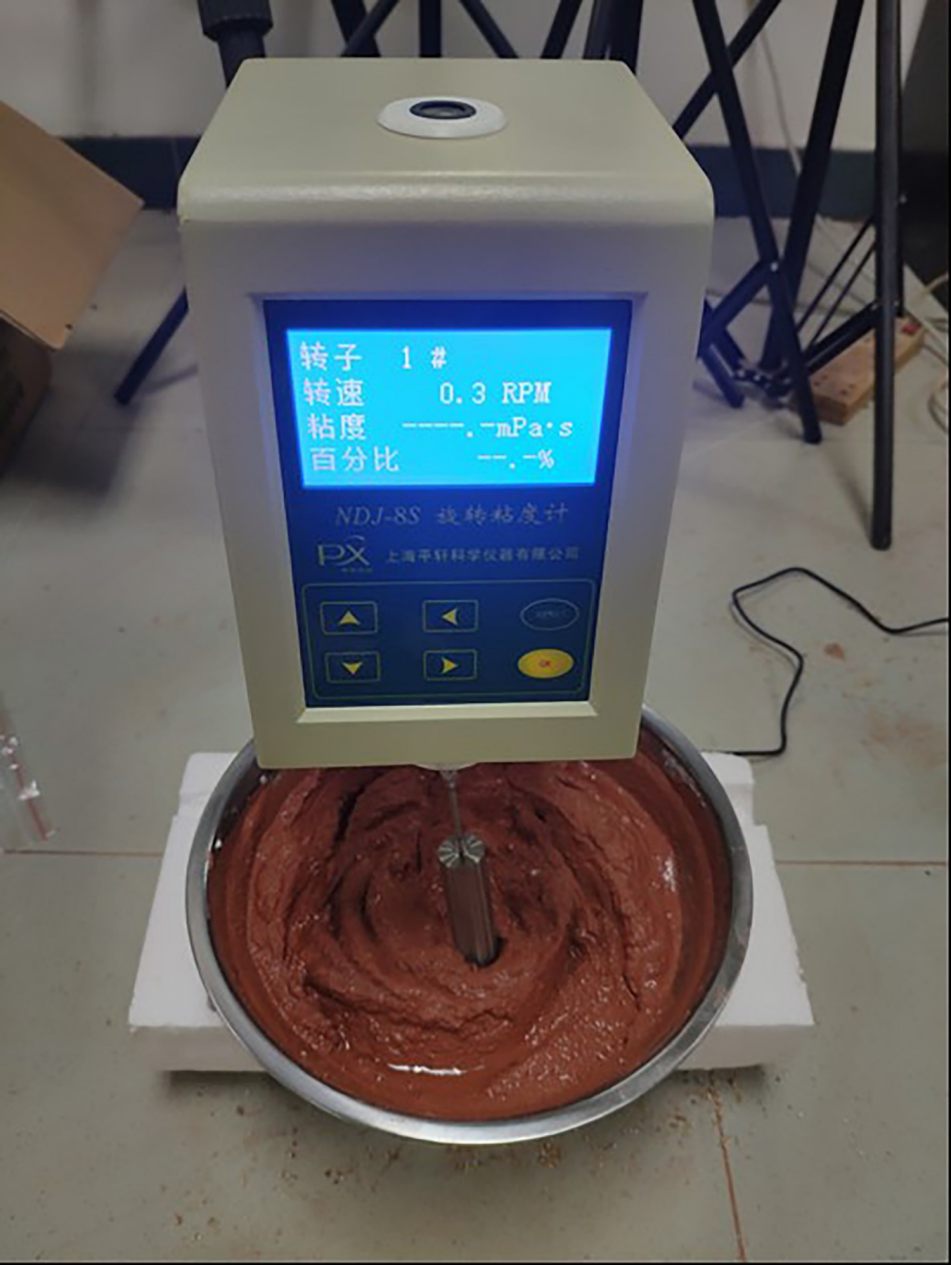
Viscosity measurement of the naturally weathered red-bed soil slurry.

Based on the principle of factorial design, the orthogonal test of three factors and three levels of weathered red-bed soil slurry is carried out to study the effects of viscosity, moisture content and particle size of weathered red-bed soil slurry on the film-forming thickness to evaluate the film-forming effect of weathered red-bed soil slurry. The naturally weathered red-bed soil is soaked and stirred with water to obtain the weathered red-bed soil slurry. The weathered red-bed soil slurry has a high viscosity, good entrainment property, and a certain plasticity and is not easy to disperse in water. According to the particle gradation of the sample, 9 of test groups are set up. The mud densities of the 9 groups of weathered red-bed soil are shown in Table 1. The mud viscosity is measured by an NDJ-8S rotary viscometer, as shown in Fig 5.

**Table 1:** Orthogonal test group for the mud film formation of the weathered red-bed soil.

Group	No. 1	No. 2	No. 3	No. 4	No. 5	No. 6	No. 7	No. 8	No. 9
< 1 mm particle content (%)	29	32	46	58	54	66	72	80	85
Moisture content (%)	30	45	40	55	50	60	35	25	15
Viscosity (Pa·s)	35.1	17.6	22.1	9.3	12.6	5.7	29.6	41.2	52.6
Density ρ (g/cm^3^)	1.98	2.25	2.62	1.75	1.86	1.94	2.31	2.35	3.82

**Fig 6:**
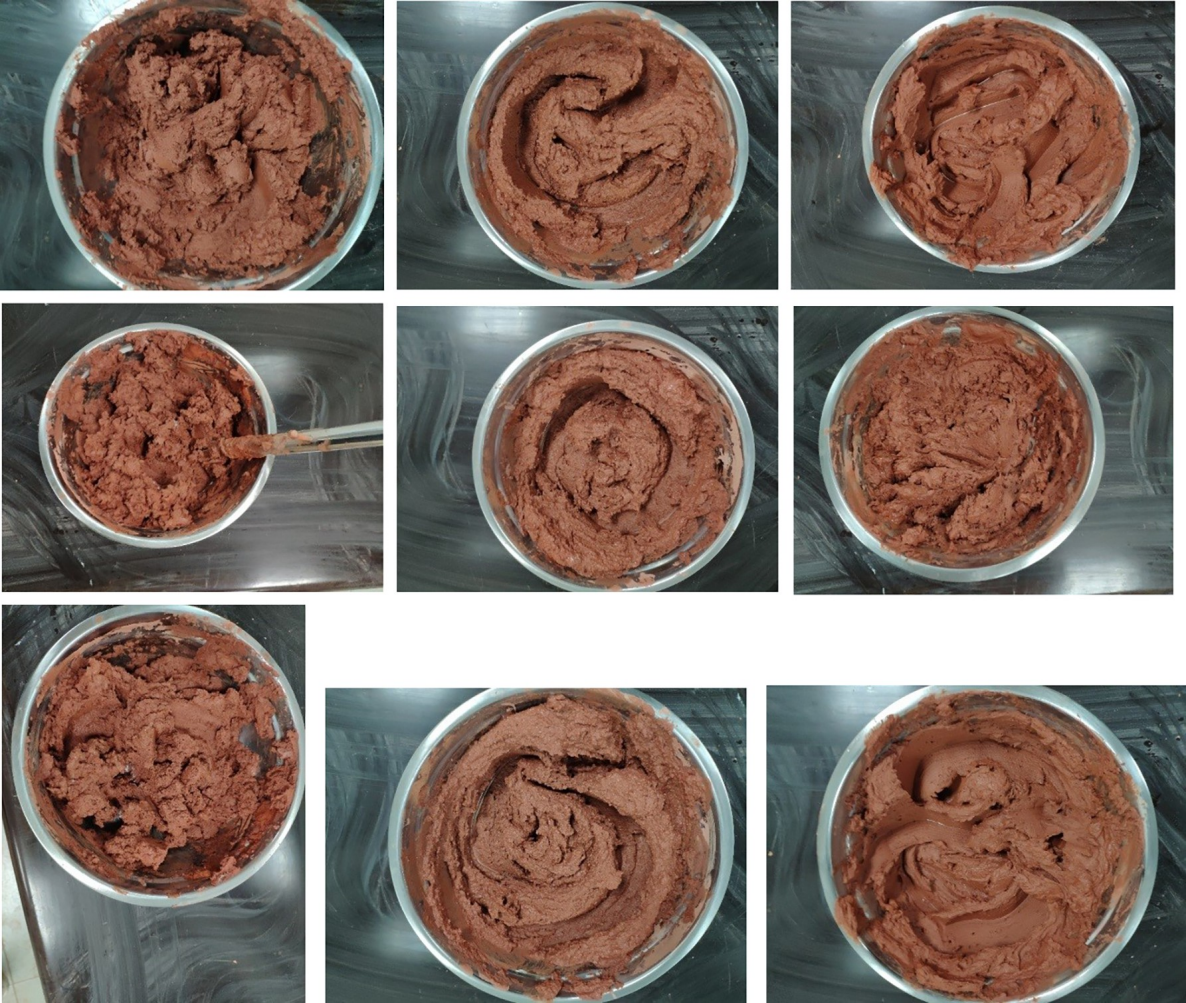
Nine naturally weathered red-bed soil slurry samples (A) No. 1; (B) No. 2; (C) No. 3; (D) No. 4; (E) No. 5; (F) No. 6 (G) No. 7; (H) No. 8; (I) No. 9.

The viscosity range of the weathered red-bed soil slurry used in the test is 5.7–52.6 Pa·s, the moisture content range is 15–60%, and the content of the particles with sizes smaller than 1 mm is 29–85%. The 9 types of slurries are shown in Fig 6.

**Fig 7:**
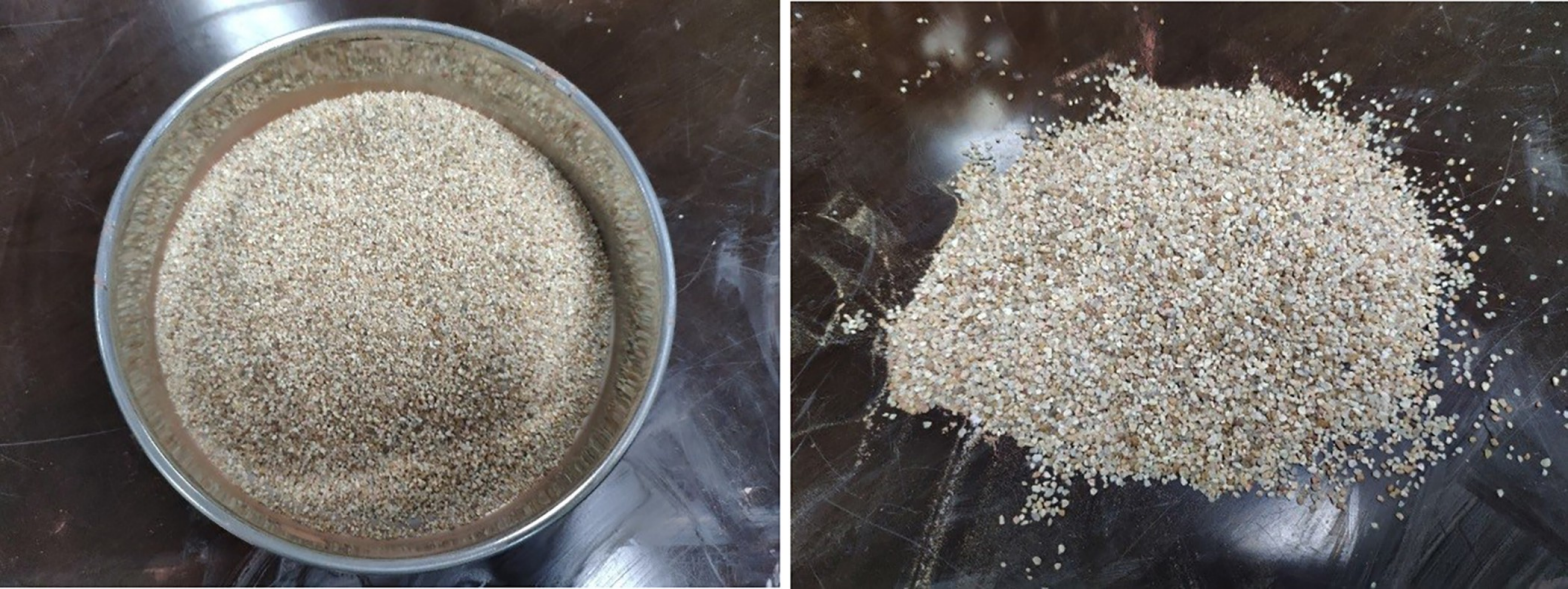
Simulated formation material.

In this test, medium-coarse sand material with a particle size range of 0.3–0.6 mm is selected as the simulated formation material. The simulated formation material is shown in Fig 7.

**Fig 8:**
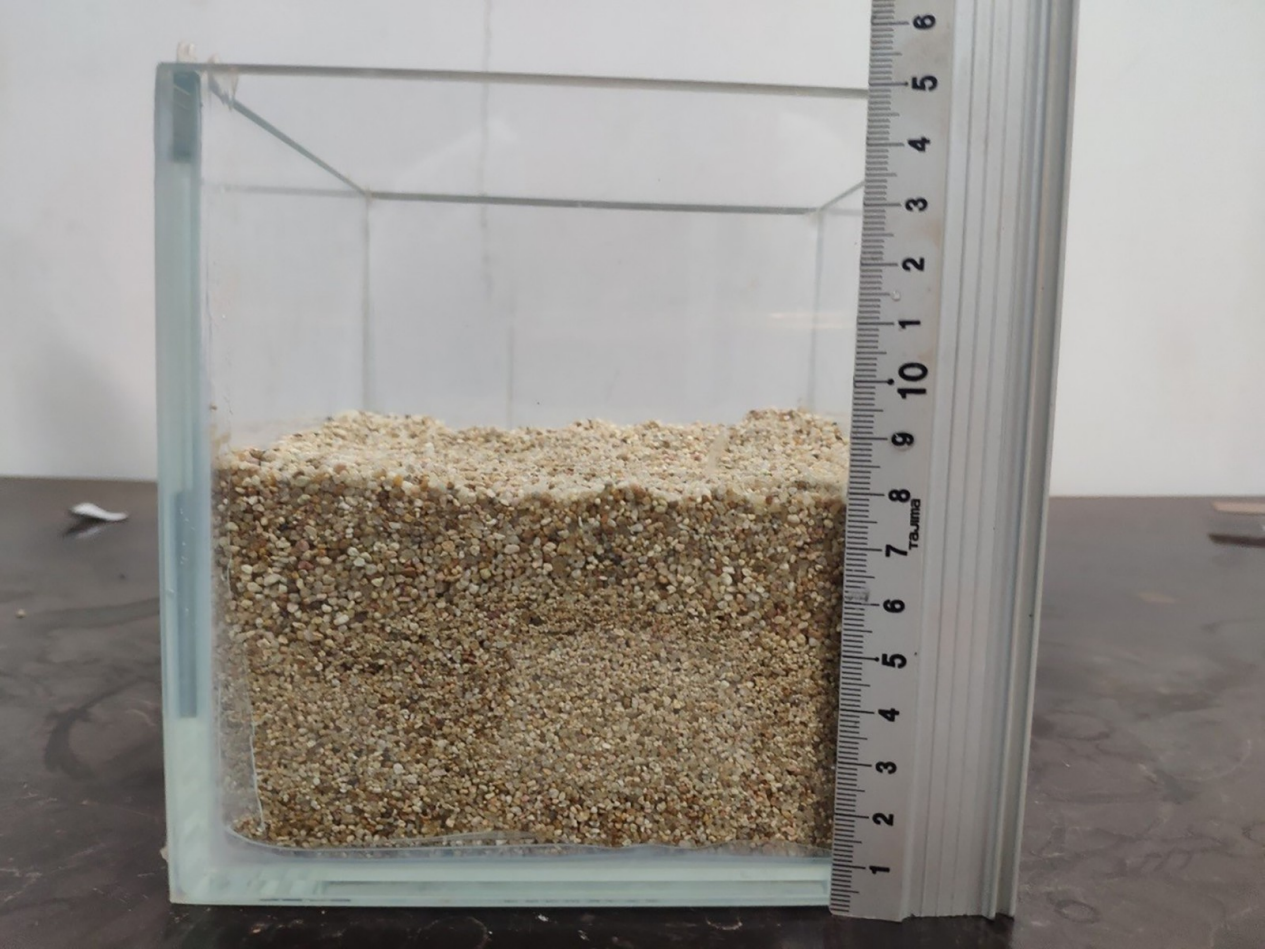
Simulated formation thickness.

The simulated stratum is placed in a transparent glass container with a length, width and height of 15 mm, and a stratum with a thickness of approximately 7–8 mm is laid in the container, as shown in Fig 8.

There are many weathered red-bed soils in nature. Red-bed soil has a very low acquisition cost and is environmentally friendly. It can quickly form a large number of high-quality films under natural conditions. Compared with the closed pressurization method, this method reduces the waste of resources and time and avoids the considerable environmental pollution caused by using artificial mud films. Thus, this test method has great practical significance.

### Mud film property test

To evaluate the properties of weathered red-bed soil films with different thicknesses, the water impermeability and acid and alkali resistances of mud films are tested. Droppers are used to continuously drip water, hydrochloric acid solution and sodium hydroxide solution. The tested concentrations of the hydrochloric acid solution and sodium hydroxide solution are 0.01 mol/L, 0.1 mol/L and 1 mol/L. The solution is dropped every 5 s for 15 min. The infiltration is recorded every 3 min. Through the infiltration of different acidic/basic solutions in the mud film, the water, acid and alkali resistances of the mud film are obtained, as shown in Table 2.

**Table 2:** Test of water, acid and alkali resistances of naturally weathered red-bed soil.

Test group	Drop solution	Dropping time (min)
W_1_	Water	3
W_2_	Water	6
W_3_	Water	9
W_4_	Water	12
W_5_	Water	15
A_1_	0.01 mol/L NaOH	3
A_2_	0.01 mol/L NaOH	6
A_3_	0.01 mol/L NaOH	9
A_4_	0.01 mol/L NaOH	12
A_5_	0.01 mol/L NaOH	15
A_6_	0.1 mol/L NaOH	3
A_7_	0.1 mol/L NaOH	6
A_8_	0.1 mol/L NaOH	9
A_9_	0.1 mol/L NaOH	12
A_10_	0.1 mol/L NaOH	15
A_11_	1 mol/L NaOH	3
A_12_	1 mol/L NaOH	6
A_13_	1 mol/L NaOH	9
A_14_	1 mol/L NaOH	12
A_15_	1 mol/L NaOH	15
S_1_	0.01 mol/L HCl	3
S_2_	0.01 mol/L HCl	6
S_3_	0.01 mol/L HCl	9
S_4_	0.01 mol/L HCl	12
S_5_	0.01 mol/L HCl	15
S_6_	0.1 mol/L HCl	3
S_7_	0.1 mol/L HCl	6
S_8_	0.1 mol/L HCl	9
S_9_	0.1 mol/L HCl	12
S_10_	0.1 mol/L HCl	15
S_11_	1 mol/L HCl	3
S_12_	1 mol/L HCl	6
S_13_	1 mol/L HCl	9
S_14_	1 mol/L HCl	12
S_15_	1 mol/L HCl	15

**Fig 9:**
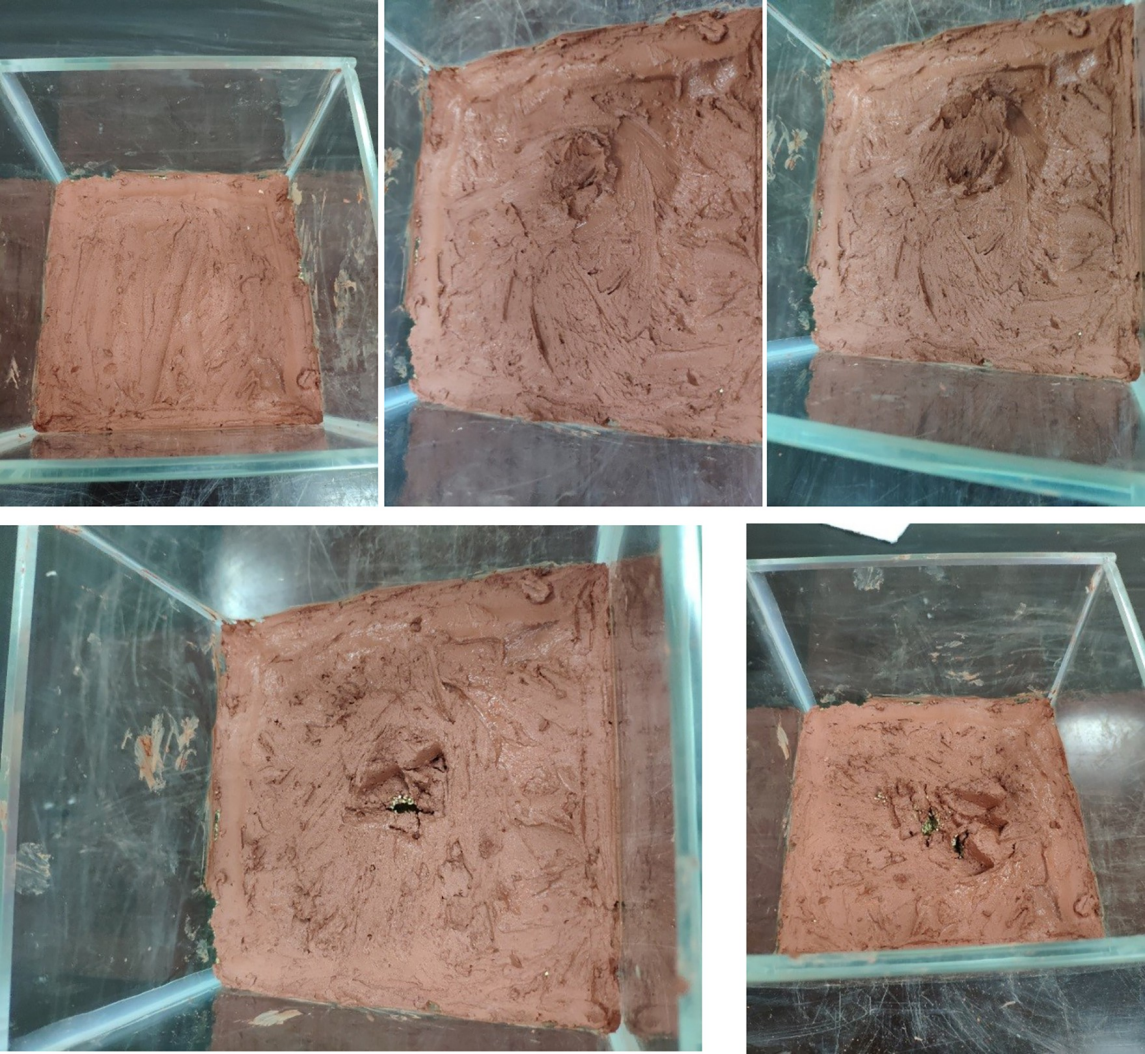
Compression test of mud film of naturally weathered red-bed soil (A) Mud film; (B) Application of 100 N pressure to mud film; (C) Application of 200 N pressure to mud film; (D) Application of 400 N pressure to mud film; (E) Application of 800 N pressure to mud film.

At the same time, to test the compression resistance of the films, pressures of 100 N, 200 N, 400 N and 800 N are applied to the mud films with different thicknesses. The pressure is maintained for 30 min, and the film damage is recorded, as shown in Fig 9.

**Fig 10:**
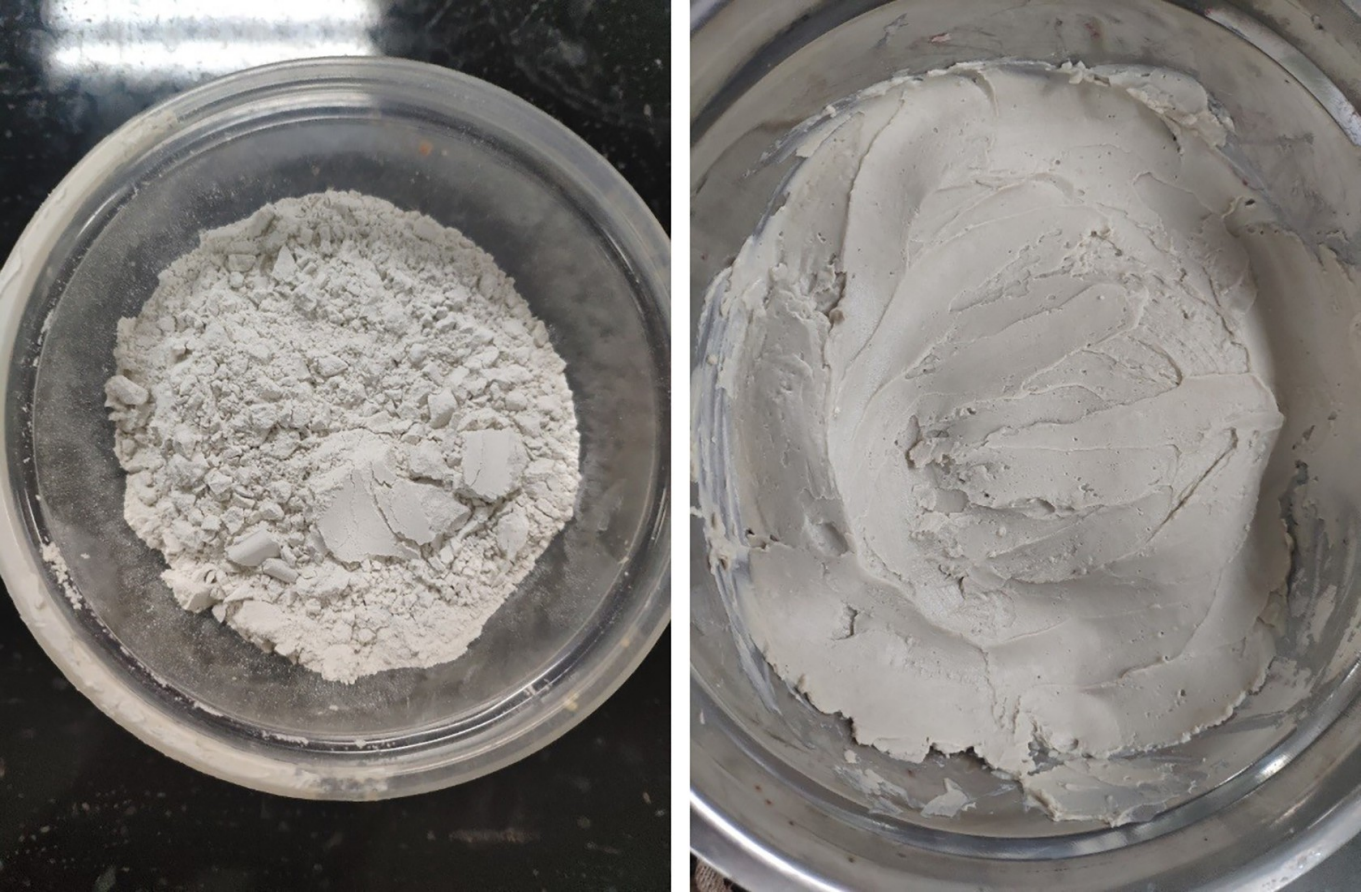
Bentonite material.

Bentonite is commonly used in mud film-forming efforts. To further explore the film-forming effect of naturally weathered red-bed soil, bentonite is mixed into mud, and the thickness, water resistance, compression resistance and acid and alkali resistances of bentonite mud film are tested. The naturally weathered red-bed soil film and the film-forming time of bentonite mud film are recorded in detail during the film-forming process, and its film-forming effect is compared with that of the naturally weathered red-bed soil and international standards, as shown in Fig 10.

## Research results and discussion

### Pressureless film-forming effect

**Fig 11:**
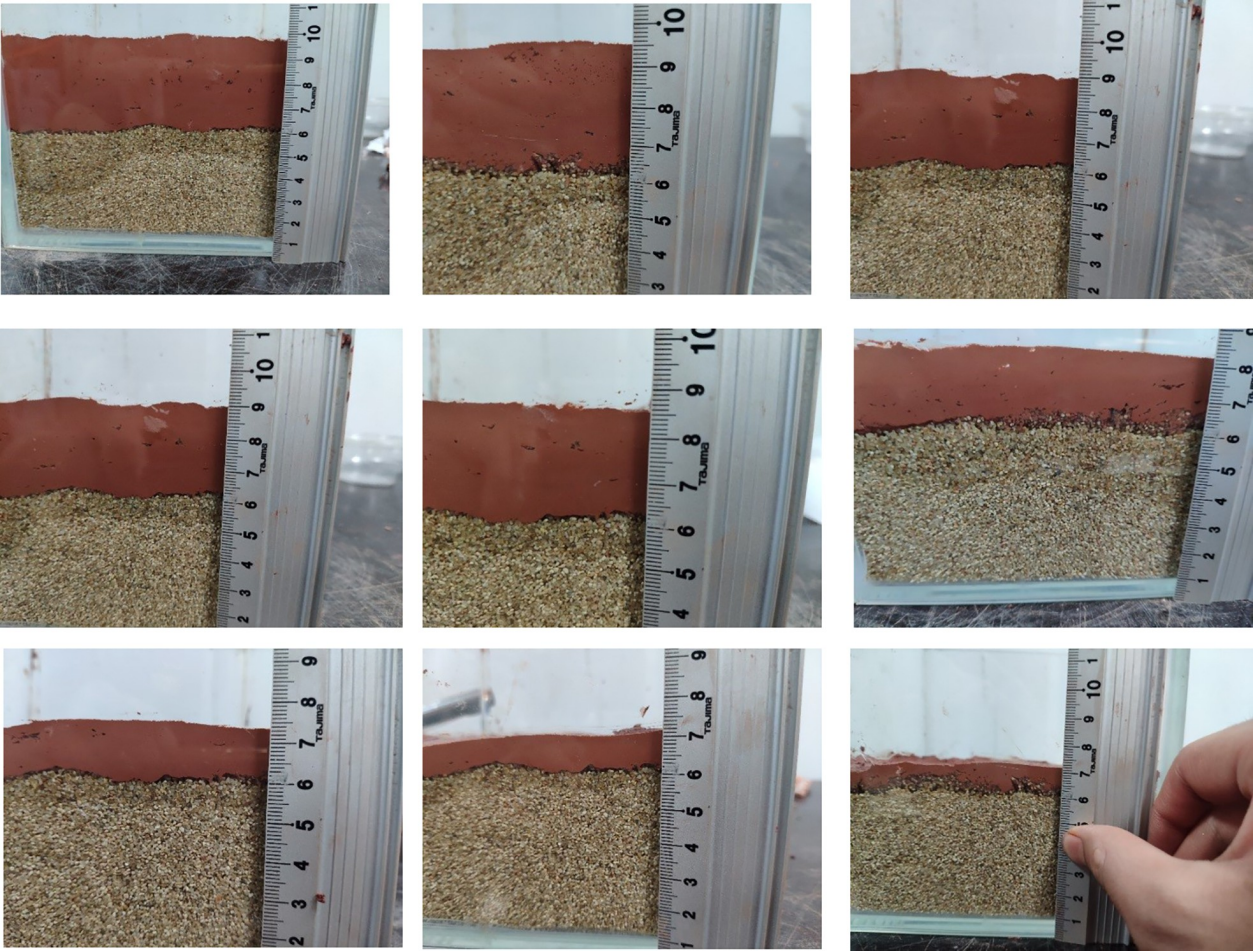
Thickness of weathered soil film for each of the 9 groups of tests (A) No. 1; (B) No. 2; (C) No. 3; (D) No. 4; (E) No. 5; (F) No. 6 (G) No. 7; (H) No. 8; (I) No. 9.

During the film-forming process of weathered red-bed soil slurry, soil particles are redistributed on the formation surface under the action of air pressure and water infiltration, which is manifested as consolidation under the action of accumulation and pressure drop. The weathered red-bed soil slurry percolates in the stratum, the soil particles are gradually separated from the water, and the water penetrates into the deeper stratum, while the soil particles block the shallow pore space to form a dense weathered red-bed soil film. According to the principle of the orthogonal test, nine groups of orthogonal tests with three factors and three levels are carried out. The nine types of mud are paved on the simulated stratum and slightly compacted to form a dense film. The thickness of the weathered red-bed soil film under different viscosities, water contents and particle contents smaller than 1 mm is studied. The thickness of the weathered soil film in group No. 1 is 37 mm; No. 2, 35 mm; No. 3, 32 mm; No. 4, 30 mm, No. 5, 25 mm; No. 6, 20 mm; No. 7, 14 mm; No. 8, 11 mm; and No. 9, 8 mm. The film formation of the 9 groups of weathered red-bed soil samples is shown in Fig 11.

The theory of mud film formation was first put forward by Ruth et al. [14] and gradually improved through the work of Grace [15], Tiller et al. [16] and Shirato et al. [17]. This theory is widely used in the chemical engineering and petroleum industries, but its application in geotechnical engineering is still in its infancy and is mainly used to predict the permeability coefficient of cut-off walls [18]. This section describes the basic assumptions and governing equations of mud film-forming theory and deduces the functions of mud film thickness with respect to particle gradation, moisture content, viscosity and density through the governing equations. Mud film-forming theory assumes that mud is a suspension composed of water and solid particles. When the mud flows into the formation in a vertical direction, the solid particles accumulate on the formation surface to form a mud film.

The water in the mud passes through the formation to form leachate. Assuming that the flow of water in the mud film conforms to Darcy’s law and that the flow rate is equal to the leachate fluid, Formula (1) can be deduced.

$$\frac{dV}{Adt}=\frac{k_{c}\Delta H}{L}=\frac{k_{c}\Delta P}{\gamma_{w}L}$$ (1)

where *V* is the volume of the leachate, *A* is the cross-sectional area of the overflow, *t* is the infiltration time, *k*_*c*_ is the average permeability coefficient of the mud film, Δ*H* is the head difference before and after mud film formation, *L* is the thickness of the mud film, *γ*_*w*_ is the gravity of the water, and Δ*P* is the pressure difference before and after mud film formation, which is equal to the pressure difference before and after the mud film forms in the filtration test.

Assuming that the volume and mass of the mud film and leachate remain unchanged after mud film formation, the volume of mud film is directly proportional to the volume of leachate. Therefore, the calculation formula of mud film thickness L can be deduced, as shown in Formula (2).

$$L=\frac{\beta V}{A}\notin$$ (2)

where coefficient *β* can be deduced according to the conservation of volume and mass, as shown in Formula (3).

$$\beta =\frac{\left(1-C)\rho_{W}(1+e\right)}{C\rho_{s}-(1-C)e\rho_{W}}$$ (3)

where *C* is the water content of the mud, *M* is the total mass of the mud, *M* is the density of solid particles in the mud, *ρ*_*W*_ is the density of the water, and *e* is the average void ratio of the mud film.

**Fig 12:**
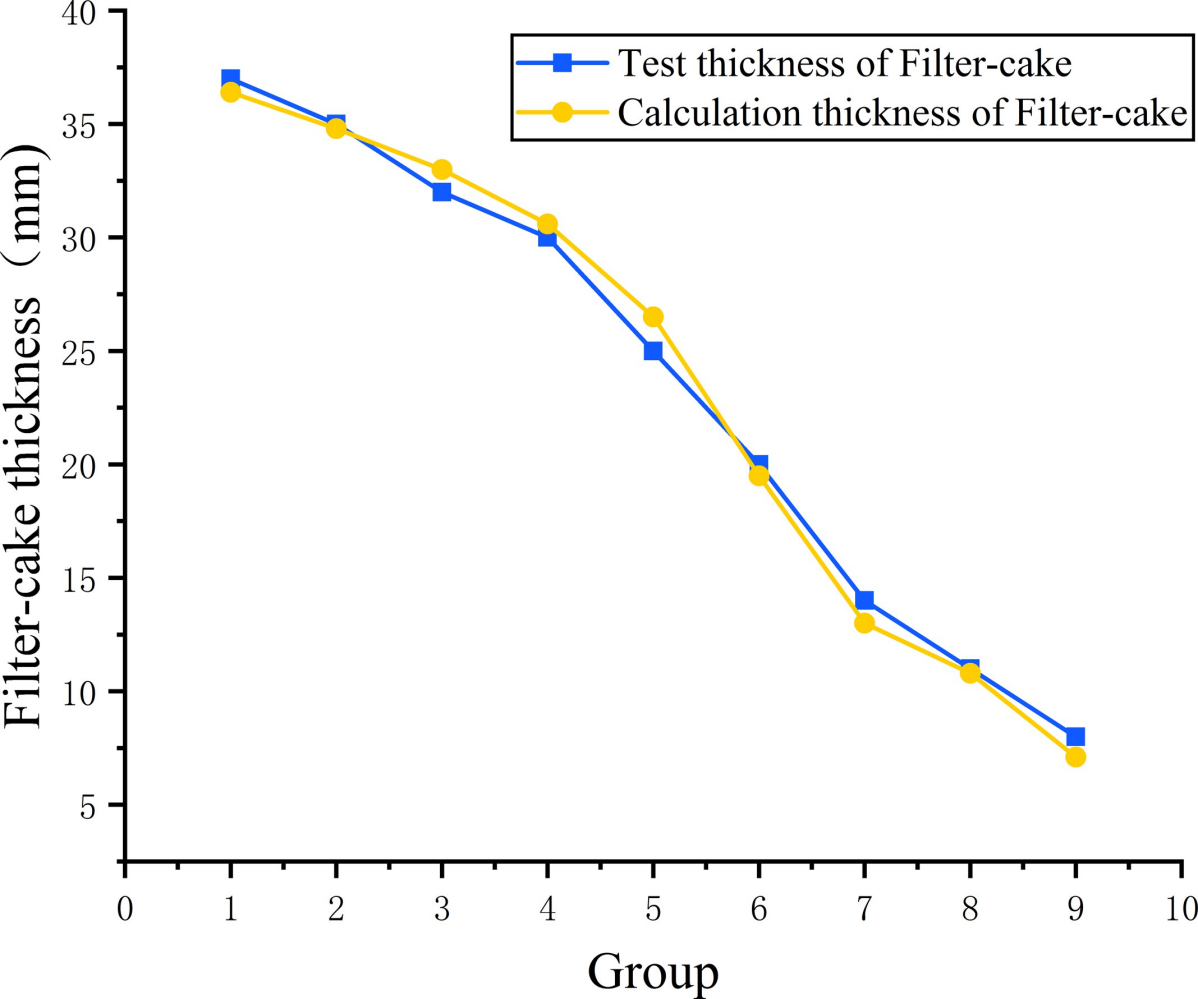
Comparison between the theoretical and experimentally obtained mud film thicknesses.

The parameters such as moisture content, density, viscosity and particle gradation of 9 groups of samples are obtained through testing. The naturally weathered red-bed soil slurry is aid in a cubic transparent glass container with a length, width and height of 15 mm, and the slurry percolation liquid volume V and overflow section area a remain unchanged. According to Formula (2) and Formula (3), the calculation of the film thickness is affected by the water content and density. Substituting the moisture content and density results of the 9 groups of samples into Formula (2) and Formula (3), Fig 12 is obtained.

As shown in Table 3, the calculated thickness of the mud film obtained by theoretical calculation is *A*, and the thickness of the mud film obtained by the test is *B*. If the relative error is *φ*, then

$$\varphi =\frac{A-B}{B}\times 100\%$$ (4)

**Table 3:** Mud film thickness error of the theoretical calculation relative to the experimentally obtained results.

Group	Calculated mud film thickness *A* (mm)	Tested mud film thickness *B* (mm)	Relative error *φ* (%)
No. 1	36.4	37	-1.6%
No. 2	34.8	35	-0.6%
No. 3	33	32	3.1%
No. 4	30.6	30	2%
No. 5	26.5	25	6%
No. 6	19.5	20	-2.5%
No. 7	13	14	-7.1%
No. 8	10.8	11	-1.8%
No. 9	7.5	8	-6.3%

The relative error between the theoretically calculated and experimentally obtained mud film thickness results is only -7.1%~6%, indicating that the mud film thickness prediction is accurate and that the mud film thickness is affected by the water content and density.

### Optimal solution of mud film formation

The influence degree of each factor and the optimal parameter combination value of mud are obtained by the orthogonal test analysis method. As shown in Table 4, the results of nine groups of tests are obtained according to the standard orthogonal table. The test results of each factor are divided into three groups, represented by T_1_, T_2_ and T_3_. The horizontal results of each factor are added, the mean value of each group is calculated, and the range of the mean value under each factor is calculated. The greater the range is, the higher the importance of the factor.

**Table 4:** Orthogonal test results of mud film formation from weathered red-bed soil.

Group	Viscosity S (Pa·s)	Moisture content ρ (%)	<1 mm particle content D (%)	Mud film thickness T (mm)
No. 1	S_1_: 35.1	ρ_1_: 30	D_1_: 29%	37
No. 2	S_1_: 17.6	ρ_2_: 45	D_2_: 32%	35
No. 3	S_1_: 22.1	ρ_3_: 40	D_3_: 46%	32
No. 4	S_2_: 9.3	ρ_3_: 55	D_1_: 58%	30
No. 5	S_2_: 12.6	ρ_1_: 50	D_2_: 54%	25
No. 6	S_2_: 5.7	ρ_2_: 60	D_3_: 66%	20
No. 7	S_3_: 29.6	ρ_2_: 35	D_1_: 72%	14
No. 8	S_3_: 41.2	ρ_3_: 25	D_2_: 80%	11
No. 9	S_3_: 52.6	ρ_1_: 15	D_3_: 85%	8
T_1_	104	70	83	/
T_2_	75	69	71	/
T_3_	33	73	60	/
T_1_ mean value	34.7	23.3	27.7	/
T_2_ mean value	25	23	23.7	/
T_3_ mean value	11	24.3	20	/
T mean range	23.7	1.3	7.7	/

**Fig 13:**
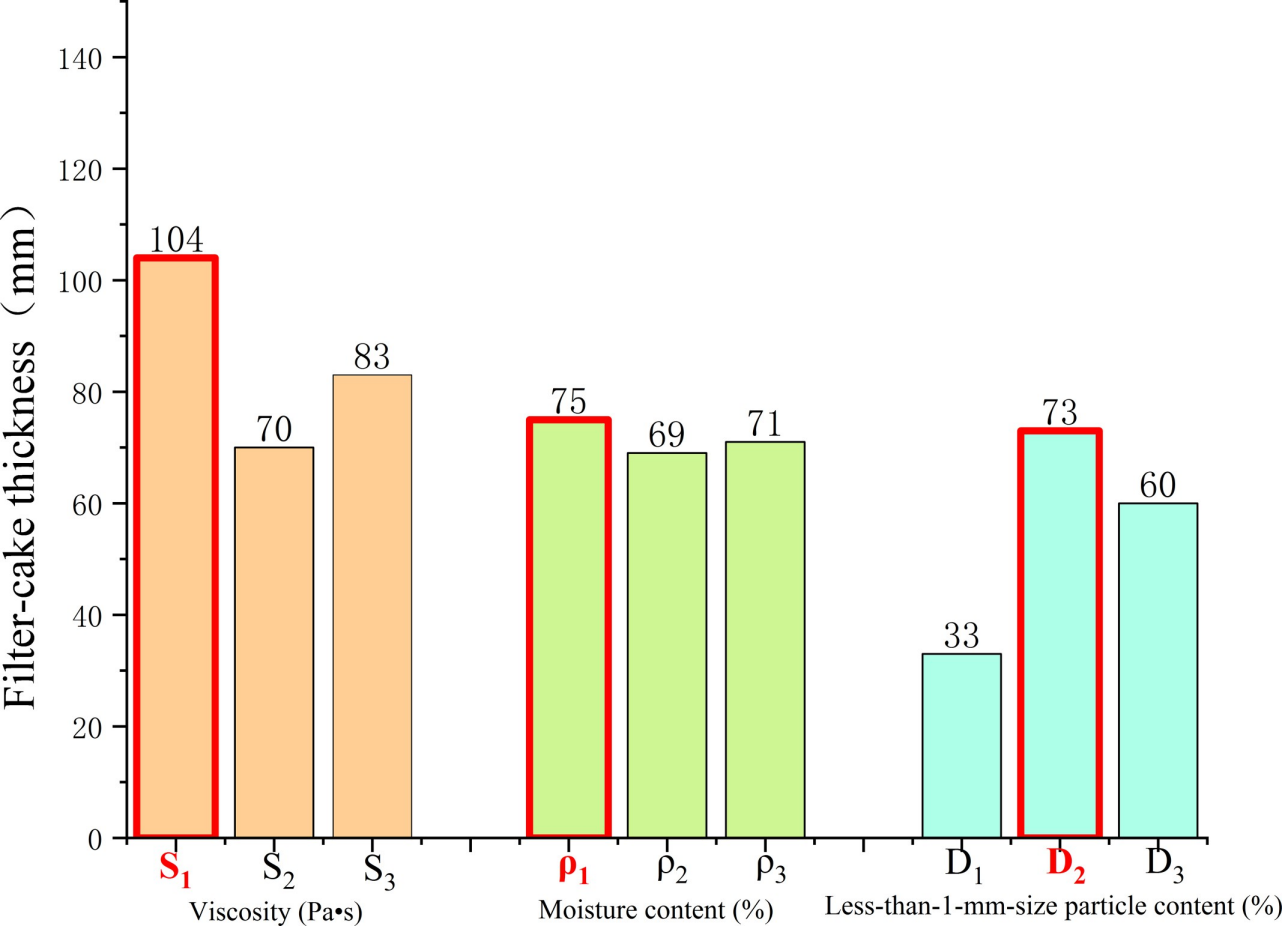
Analysis of the influence of mud film thickness on weathered red-bed soil.

The average value of each factor is shown in Fig 13. The influences on the thickness of the mud film of weathered red-bed soil are ranked as follows: the viscosity has the largest influence, the water content follows, and the particle content smaller than 1 mm has the smallest influence. The best parameter values for film forming of weathered red-bed soil are as follows: viscosity of 29.6–52.6 Pa·s, moisture content of 15–35%, and particle content smaller than 1 mm of 75%-85%.

### Influence of mud properties on film-forming effect

**Fig 14:**
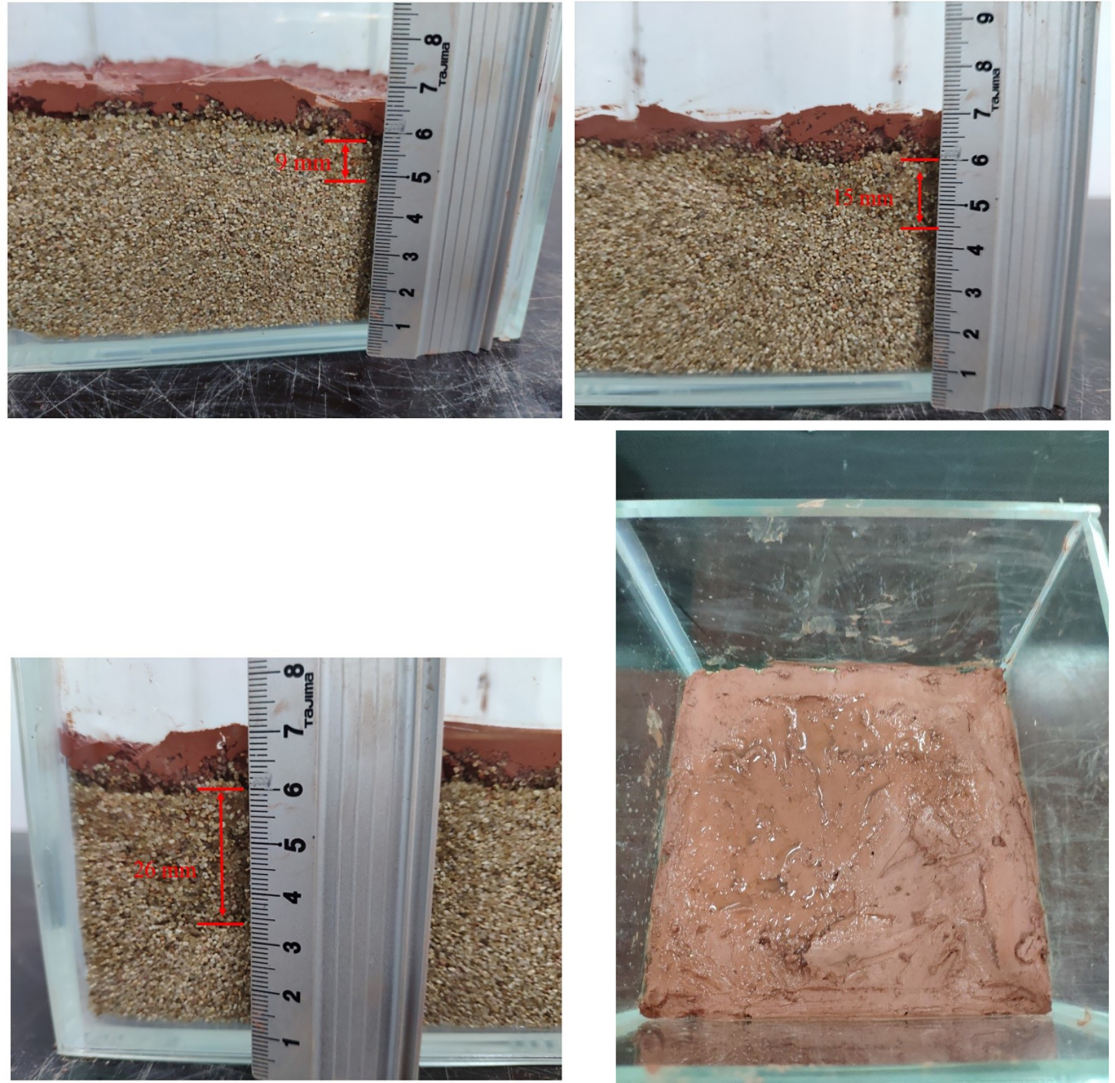
Infiltration of 8 mm thick naturally weathered red-bed soil films after dropping water (A) Water dropped on an 8 mm thick naturally weathered red-bed soil film for 3 m; (B) Water dropped on an 8 mm thick naturally weathered red-bed soil film for 6 min; (C) Water dropped on an 8 mm thick naturally weathered red-bed soil film for 9 min; (D) Water dropped on an 8 mm thick naturally weathered red-bed soil film for 12 min.

**Fig 15:**
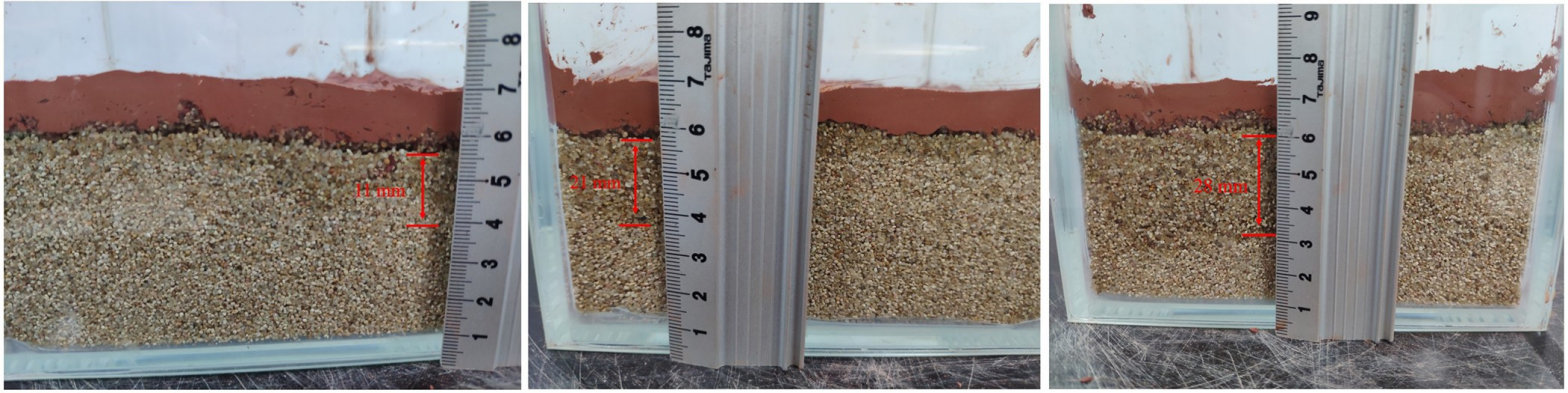
Infiltration of 11 mm thick naturally weathered red-bed soil films after dropping 0.1 mol/L NaOH (A) 0.1 mol/L NaOH dropped on an 11 mm thick naturally weathered red-bed soil film for 3 min; (B) 0.1 mol/L NaOH dropped on an 11 mm thick naturally weathered red-bed soil film for 6 min; (C) 0.1 mol/L NaOH dropped on an 11 mm thick naturally weathered red-bed soil film for 9 min.

**Fig 16:**
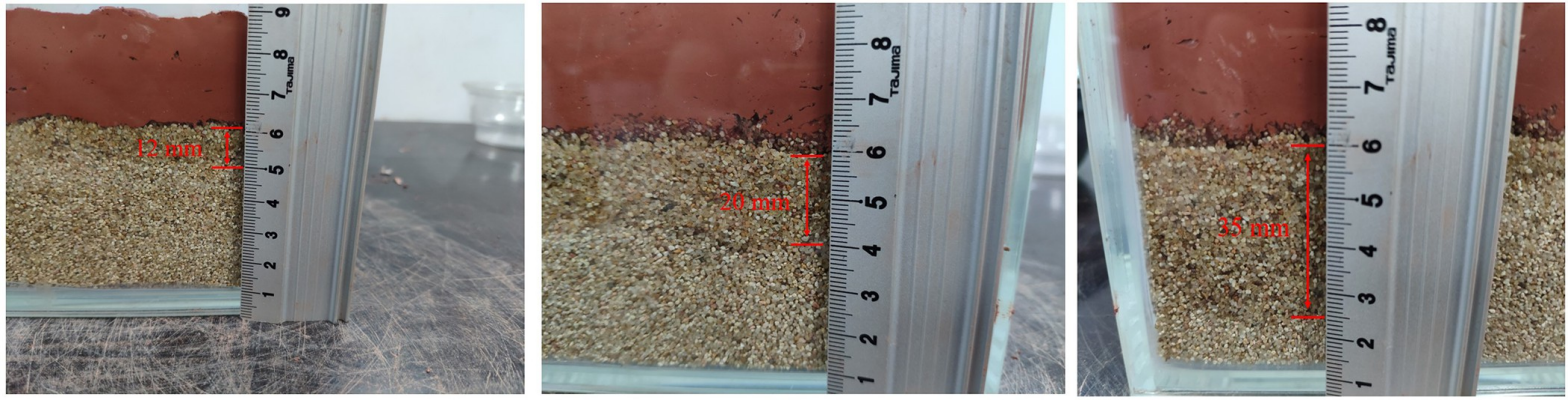
Infiltration of 32 mm thick naturally weathered red-bed soil films after dropping 1 mol/L HCl (A) 1 mol/L HCl dropped on a 32 mm thick naturally weathered red-bed soil film for 3 min; (B) 1 mol/L HCl dropped on a 32 mm thick naturally weathered red-bed soil film for 6 min; (C) 1 mol/L HCl dropped on a 32 mm thick naturally weathered red-bed soil film for 9 min.

The nature of mud film is important not only to know for subsequent ecological restoration but also to evaluate the effect of film formation. Through dripping water, hydrochloric acid solution and sodium hydroxide solution onto the naturally weathered red-bed soil film, its water impermeability and acid and alkali resistances are evaluated. The infiltration phenomenon is shown in Figs 14–16.

**Fig 17:**
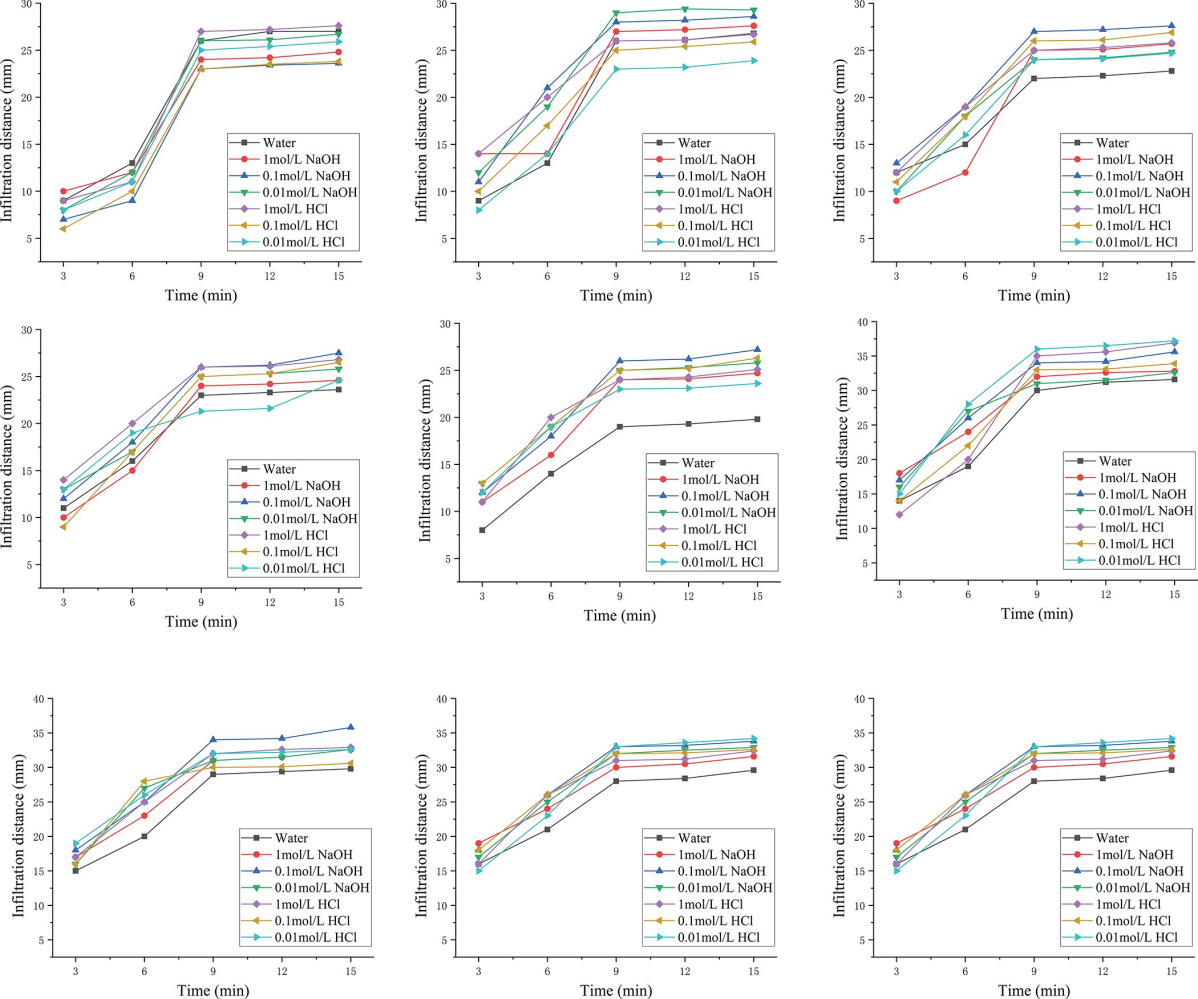
Infiltration of the mud film of weathered soil in the 9 groups (A) 8 mm naturally weathered red-bed soil film; (B) 11 mm naturally weathered red-bed soil film; (C) 14 mm naturally weathered red-bed soil film; (D) 20 mm naturally weathered red-bed soil film; (E) 25 mm naturally weathered red-bed soil film; (F) 30 mm naturally weathered red-bed soil film; (G) 32 mm naturally weathered red-bed soil film; (H) 35 mm naturally weathered red-bed soil film; (I) 8 mm naturally weathered red-bed soil film.

As shown in Fig 17, the solution is dropped onto the film, and the infiltration distance of the solution on the simulated formation over time is recorded. The 15 min infiltration distance of the 9 groups of samples is 6–35.6 mm. With the addition of water and different concentrations of hydrochloric acid, the difference in film infiltration distance after sodium hydroxide solution is within 5%, indicating that the acid and alkali resistances of the naturally weathered red-bed soil film are good and that pH has little effect on the water impermeability of weathered red-bed soil films.

After 9 min, the infiltration distances of films with different thicknesses tend to be stable, the infiltration distance will not increase, and there will be liquid on the film surface, indicating that at this time, the film infiltration reaches stability, and the solution will no longer penetrate into the simulated formation. Thus, the formation will exhibit a strong water resistance after reaching permeability equilibrium. The infiltration distance of the solution in the stratum for the 30 mm thick film is greater than that of the 10–20 mm films. Reducing the film thickness will not weaken the water impermeability of the film, but the film thickness can meet the needs of slope support. Therefore, a film of approximately 10 mm has strong practicability.

**Fig 18:**
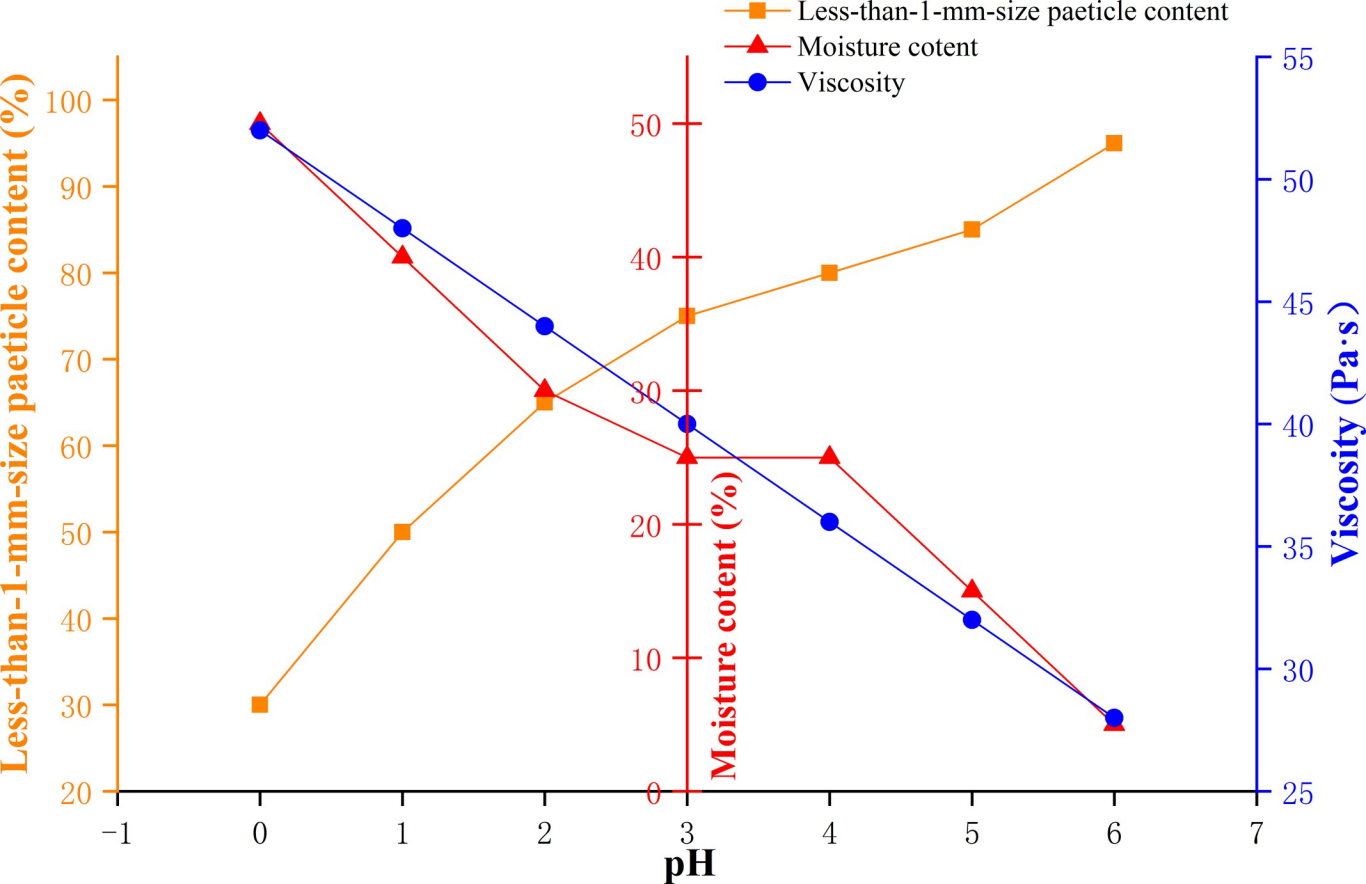
Effects of mud viscosity, water content and particle content smaller than 1 mm on the acid resistance of a mud film.

**Fig 19:**
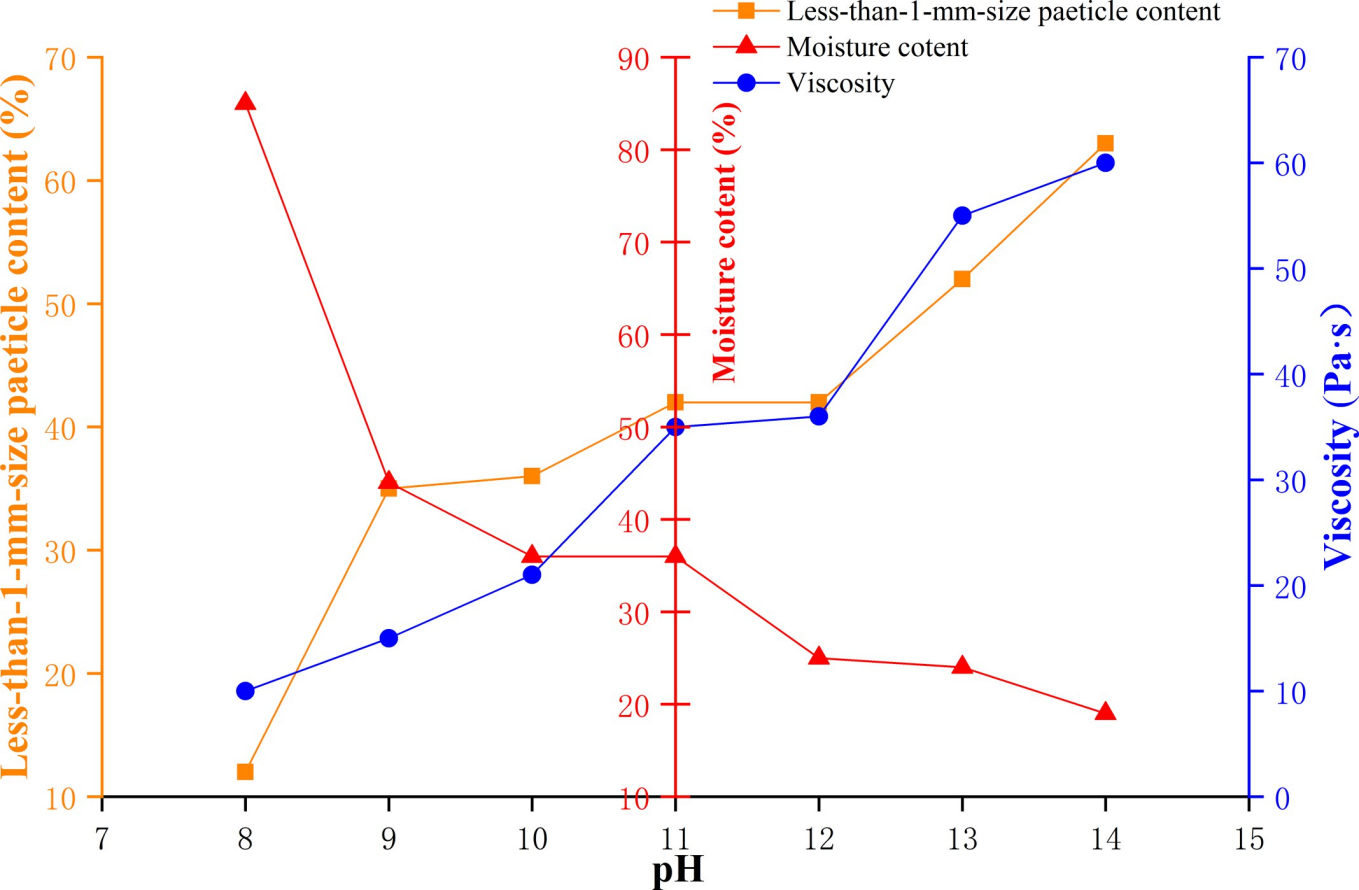
Effects of mud viscosity, water content and particle content smaller than 1 mm on the alkaline resistance of a mud film.

**Fig 20:**
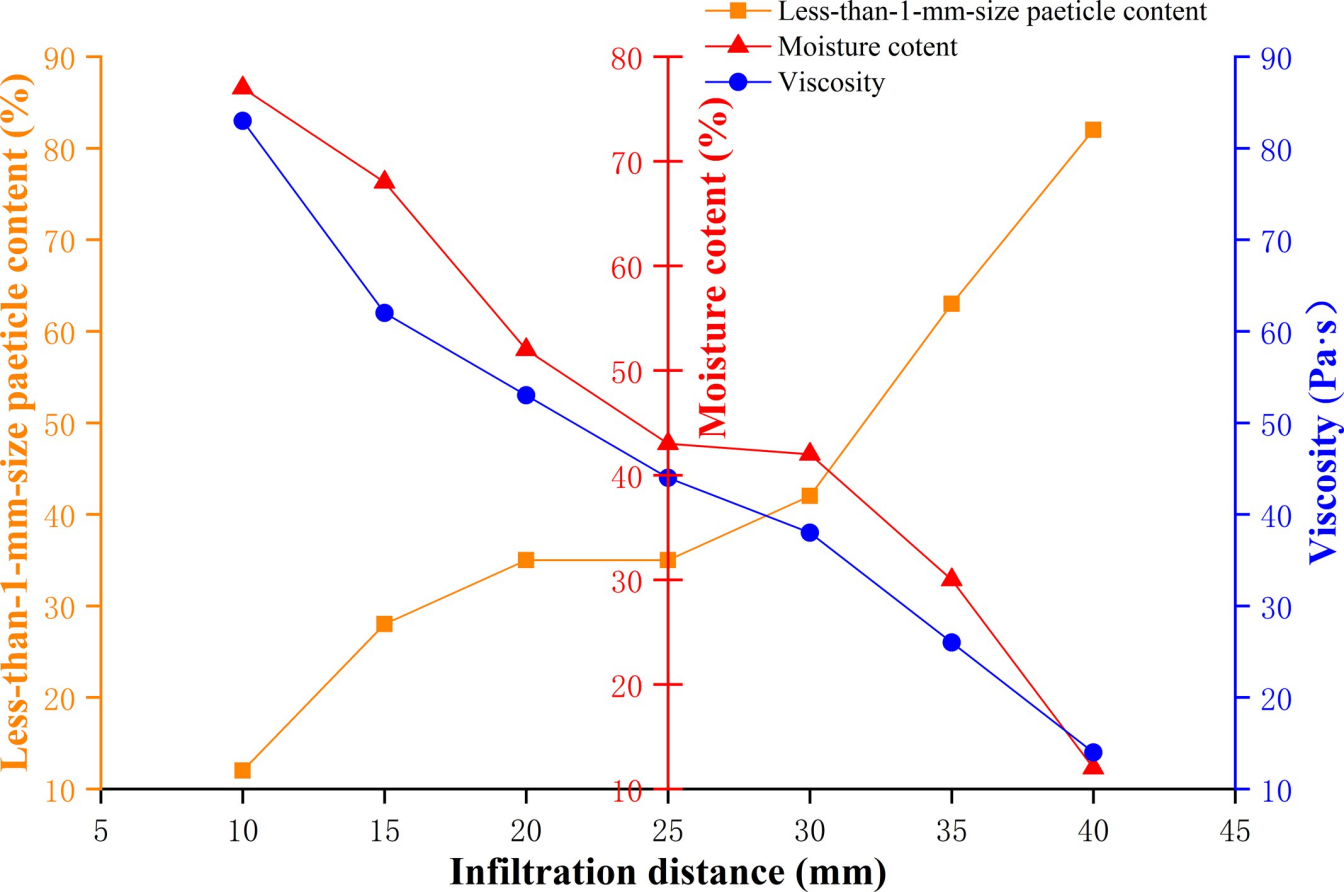
Effects of mud viscosity, water content and particle content smaller than 1 mm on the infiltration distance of a mud film.

The influences of a mud viscosity of 29.6–52.6 Pa·s, moisture content of 15–35%, and particle content smaller than 1 mm of 75%-85% on the acid resistance, alkali resistance and infiltration distance of the naturally weathered red-bed soil-derived mud films are shown in Figs 18–20.

As shown in Fig 18, when the particle content smaller than 1 mm increases gradually, the acid resistance of the mud film decreases gradually; when the viscosity and water content of the mud increase gradually, the acid resistance of the mud film increases gradually. The mud film has the strongest acid resistance when the mud viscosity of the naturally weathered red-bed soil is 53 Pa·s, the water content is 50%, and the particle content smaller than 1 mm is 30%. When the mud viscosity of naturally weathered red-bed soil is 28 Pa·s, the water content is 5%, and the particle content smaller than 1 mm is 95%, the acid resistance of the resulting mud film is the weakest.

As shown in Fig 19, when the water content of the mud increases gradually, the alkali resistance of the resulting mud film decreases gradually. When the mud viscosity and particle content smaller than 1 mm increase gradually, the alkali resistance of the mud film increases gradually. The alkaline resistance is the strongest when the mud viscosity of naturally weathered red-bed soil is 60 Pa·s, the water content is 20%, and the particle content smaller than 1 mm is less than 60%. For naturally weathered red-bed soil, when the mud viscosity is 10 Pa·s, the moisture content is 88%, and the particle content smaller than 1 mm is 12%, the alkaline resistance of the mud film is the weakest.

As shown in Fig 20, when the particle content smaller than 1 mm increases gradually, the mud film infiltration distance gradually increases. When the viscosity and water content of the mud increase gradually, the infiltration distance of the mud film decreases gradually. When the mud viscosity of the naturally weathered red-bed soil is 14 Pa·s, the water content is 12%, and the particle content smaller than 1 mm is 80%, the mud film infiltration distance is the longest. When the mud viscosity of the naturally weathered red-bed soil is 85 Pa·s, the water content is 79%, and the particle content smaller than 1 mm is 12%, the mud film infiltration distance is the smallest.

The 8 mm film (No. 9) will break after being subjected to 800 N pressure, and the stratum will expose the surface, while the 11 mm film (No. 8) will not break after being subjected to 800 N pressure, which can ensure the integrity of the film structure, indicating that the weathered red-bed soil film has strong compression resistance at 11 mm, which can reduce the thickness and ensure the film quality. As shown in Table 5.

**Table 5:** Comparison of the compression resistances of the 8 mm film and 11 mm film.

Apply pressure (N)	11 mm naturally weathered red-bed soil film	8 mm naturally weathered red-bed soil film
100	No significant change	No significant change
200	No significant change	Slight rupture
400	No significant change	Obvious rupture
800	No significant change	Severe rupture

Bentonite is a common mud film-forming material. The properties of the naturally weathered red-bed soil films were compared with those of an 11 mm bentonite film to evaluate the universality of the naturally weathered red-bed soil film.

**Fig 21:**
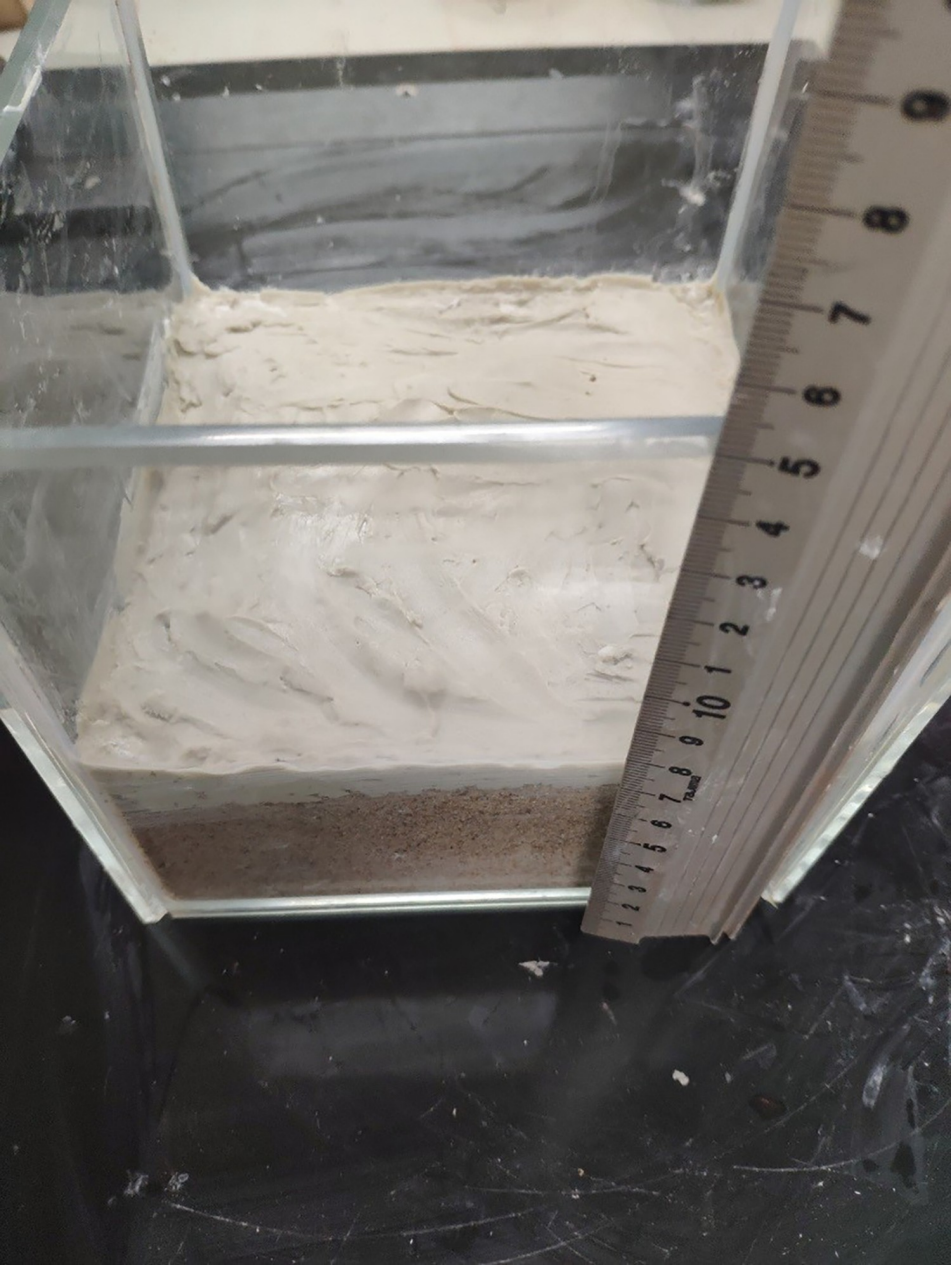
11 mm bentonite film.

For this, the same coarse sand is placed in the glass container, and the bentonite slurry is gently pressed into an 11 mm film by tiling, as shown in Fig 21.

**Fig 22:**
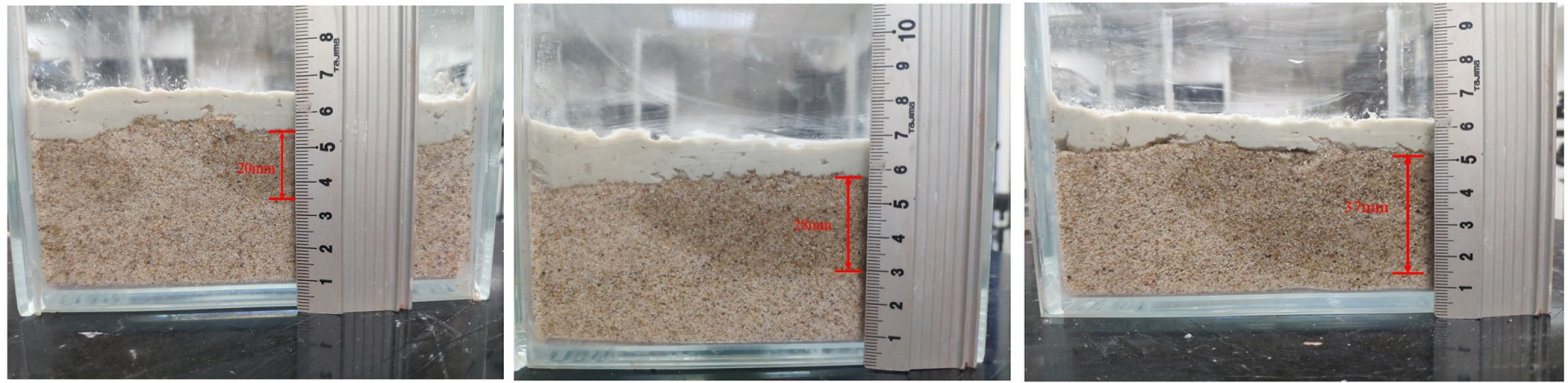
Infiltration of 11 mm thick bentonite films after dropping water. (A) Water dropped on an 11 mm thick bentonite film for 3 min; (B) Water dropped on an 11 mm thick bentonite film for 6 min; (C) Water dropped on an 11 mm thick bentonite film for 6 min.

The water impermeability of the 11 mm bentonite film is compared with that of the naturally weathered red-bed soil film, as shown in Table 6. The infiltration phenomenon of the 11 mm bentonite film with water and hydrochloric acid and sodium hydroxide solutions of different concentrations is shown in Fig 22.

**Table 6:** Comparison of properties between 11 mm bentonite films and naturally weathered red-bed soil films.

Test group	11 mm naturally weathered red-bed soil film infiltration distance (mm)	11 mm bentonite film infiltration distance (mm)
W_1_	9	20
W_2_	13	28
W_3_	26	37
W_4_	26.1	39.2
W_5_	26.8	41.6
A_1_	12	18
A_2_	19	26
A_3_	29	35.2
A_4_	29.4	36.3
A_5_	29.3	37.2
A_6_	11	17
A_7_	21	24.6
A_8_	28	33.8
A_9_	28.2	35.2
A_10_	28.6	36.5
A_11_	14	16.2
A_12_	14	23.5
A_13_	27	31.5
A_14_	27.2	32.4
A_15_	27.6	33.6
S_1_	8	18.6
S_2_	14	24.1
S_3_	23	32.7
S_4_	23.2	33.5
S_5_	23.8	34.1
S_6_	10	17.8
S_7_	17	24.3
S_8_	25	30.5
S_9_	25.4	31.7
S_10_	25.9	32.6
S_11_	14	15.2
S_12_	20	21.8
S_13_	26	31.9
S_14_	26.1	32.6
S_15_	26.7	32.8

After the water, hydrochloric acid solution and sodium hydroxide solution are added dropwise, the infiltration distances of the naturally weathered red-bed soil film are 14–29.4 mm, and those of the bentonite film are 17–41.6 mm. Both types of films are stable after 9 min of fluid dropping. There is little difference between the infiltration distances of an acidic/basic solution and water. The hydrochloric acid solution and sodium hydroxide solution have little effect on the water impermeability of the two films, and both films can resist them. The naturally weathered red-bed soil film has a smaller infiltration distance and shows better water resistance than the bentonite film does, but both 11 m films have good compression resistance. Unlike bentonite, naturally weathered red-bed soil is widely distributed in nature, easy to obtain, inexpensive, and environmentally friendly and thus has very strong practicability and feasibility for producing films similar to those of bentonite films.

Therefore, the best thickness of weathered red-bed soil films in terms of water resistance and compression resistance is 11 mm, and the best parameters for film formation are a mud viscosity of 41.2 Pa·s, moisture content of 25%, and particle content smaller than 1 mm of 80%. Compared with the 11 mm bentonite film, this weathered red-bed soil film not only can maintain good water resistance, compression resistance, acid resistance and alkali resistance but also may be a green, environmentally friendly product that and reduces project costs.

### Comparison of naturally weathered red-bed soil mud films with bentonite film and the international standard

In ecological restoration, the main functions of weathered red-bed soil mud film are water separation, entropy retention, acid and alkali resistance and anti-trampling, to achieve the effects of desert greening and slope restoration. According to the international ecological restoration standard <ISO 11074-4-2001> [19], the properties of naturally weathered red-bed soil mud film and bentonite mud film are compared with the international standard, and Table 7 is obtained.

**Table 7:** Comparison of ecological restoration standards.

Name	Water separation	Acid resistance	Alkali resistance	Compression	Film-forming time	Cost
Weathered red-bed soil mud film	Liquid infiltration distance <29.4 mm	Applicable scope 0<pH<7	Applicable scope 7<pH<14	Resistant to pressure >800 N	40 min	$0.5/kg
Bentonite mud film	Liquid infiltration distance <41.6 mm	Applicable scope 4<pH<7	Applicable scope 9<pH<14	Resistant to pressure >600 N	20 min	$8/kg
International standard	Liquid infiltration distance <50 mm	Applicable scope 4.5<pH<7	Applicable scope 7<pH<9.5	Resistant to pressure >600 N	Undefined	Undefined

The water impermeability of the weathered red-bed soil mud film is 30% higher than that of the bentonite mud film and 30% higher than that of the international standard; Compared with those of the bentonite mud film and international standard, the acid resistance of the weathered red-bed soil mud film is 3 pH lower, and the alkali resistance is 4 pH higher. Compared with those of the bentonite mud film and international standard, the ranges of acid and alkali resistance of the weathered red-bed soil mud film are significantly wider. Compared with those of the bentonite mud film and international standard, the compression resistance of weathered red-bed soil mud film is increased by 25%. Thus, the compressive effect is significantly enhanced. Compared with that of the bentonite mud film, the film-forming time of the weathered red-bed soil mud film is 50% longer, and the film-forming speed and construction efficiency are significantly accelerated. Compared with that of the bentonite mud film, the cost of the weathered red-bed soil mud film is 94% less, greatly reduced. The properties of weathered red-bed soil mud film meet the requirements of ecological restoration, and the effect of weathered red-bed soil mud film is better than that of common bentonite mud film. Therefore, weathered red-bed soil mud film can be used as a mud film for ecological restoration.

## Conclusion

1. Taking pure naturally weathered red-bed soil as the test material, this paper designs and carries out a mud film-forming test of naturally weathered red-bed soil under pressureless conditions and a comparison test with bentonite; tests the influence of mud particle gradation, viscosity and moisture content on the film-forming effect; optimizes the mud film-forming ratio; and further studies the thickness, water resistance, compression resistance, and acid and alkali resistances. The results show that this naturally weathered red-bed soil mud can form a film under pressureless conditions. The optimal (high-quality) film corresponds to a mud viscosity of 29.6–52.6 Pa·s, water content of 15–35%, and content of particles with particle sizes smaller than 1 mm of 75%-85%.
2. In this paper, the natural weathered red-bed clay film is compared with the international standard and bentonite clay film. The natural weathered red-bed clay film meets the ecological restoration standard. It has a better water resistance, compression resistance, acid resistance and alkali resistance than bentonite clay film. It has the advantages of a naturally wide distribution, fast film-forming speed, low cost, simple sample preparation process and wide application range. Therefore, due to its obvious advantages, weathered red-bed soil mud film can be introduced into the field of ecological restoration and has broad prospects.
3. The weathered red-bed soil mud films studied in this paper can not only be introduced into the field of ecological restoration but also be applied to the field of industrial architecture by optimizing the film-forming method to reduce the difficulty of engineering construction and increase the applications in industrial architecture design. We can also further explore whether other types of mud have similar properties and increase the breadth of mud film-forming ecological restoration.

## Supporting information

S1 File (XLSX)

S2 File (XLSX)

S3 File (XLSX)

S4 File (XLSX)

S5 File (XLSX)

S6 File (XLSX)

S7 File (XLSX)

S8 File (XLSX)

S9 File (XLSX)

S10 File (XLSX)

S11 File (XLSX)
